# Charged particle single nanometre manufacturing

**DOI:** 10.3762/bjnano.9.266

**Published:** 2018-11-14

**Authors:** Philip D Prewett, Cornelis W Hagen, Claudia Lenk, Steve Lenk, Marcus Kaestner, Tzvetan Ivanov, Ahmad Ahmad, Ivo W Rangelow, Xiaoqing Shi, Stuart A Boden, Alex P G Robinson, Dongxu Yang, Sangeetha Hari, Marijke Scotuzzi, Ejaz Huq

**Affiliations:** 1 Oxford Scientific Consultants Ltd, 67 High Street, Dorchester-on-Thames, OX10 7HN, UK; 2 Department of Imaging Physics, Delft University of Technology, Lorentzweg 1, 2628 CJ Delft, Netherlands; 3 Department of Micro- and Nanoelectronic Systems, Ilmenau University of Technology, Max-Planck-Ring 1, Ilmenau 98693, Germany; 4 Electronics and Computer Science, University of Southampton, Southampton, SO17 1BJ, UK; 5 School of Chemical Engineering, University of Birmingham, Edgbaston, Birmingham B15 2TT, UK; 6 School of Physics and Astronomy, University of Birmingham, Birmingham, Edgbaston, Birmingham, B15 2TT, UK

**Keywords:** charged particle beams, electron, field emission, ion, nanolithography

## Abstract

Following a brief historical summary of the way in which electron beam lithography developed out of the scanning electron microscope, three state-of-the-art charged-particle beam nanopatterning technologies are considered. All three have been the subject of a recently completed European Union Project entitled “Single Nanometre Manufacturing: Beyond CMOS”. Scanning helium ion beam lithography has the advantages of virtually zero proximity effect, nanoscale patterning capability and high sensitivity in combination with a novel fullerene resist based on the sub-nanometre C_60_ molecule. The shot noise-limited minimum linewidth achieved to date is 6 nm. The second technology, focused electron induced processing (FEBIP), uses a nozzle-dispensed precursor gas either to etch or to deposit patterns on the nanometre scale without the need for resist. The process has potential for high throughput enhancement using multiple electron beams and a system employing up to 196 beams is under development based on a commercial SEM platform. Among its potential applications is the manufacture of templates for nanoimprint lithography, NIL. This is also a target application for the third and final charged particle technology, viz. field emission electron scanning probe lithography, FE-eSPL. This has been developed out of scanning tunneling microscopy using lower-energy electrons (tens of electronvolts rather than the tens of kiloelectronvolts of the other techniques). It has the considerable advantage of being employed without the need for a vacuum system, in ambient air and is capable of sub-10 nm patterning using either developable resists or a self-developing mode applicable for many polymeric resists, which is preferred. Like FEBIP it is potentially capable of massive parallelization for applications requiring high throughput.

## Review

### 1 Introduction

Methods for nanostructuring – the fabrication of structures with sizes on the nanoscale – are required for the fabrication of next-generation nanoelectronics, such as quantum-based devices, for nanophotonics, nanobiotechnology, nanomaterials and nano-electro-mechanical systems. Until now, the leading method for scaled-up fabrication of nanostructures has been optical lithography, combined with pattern transfer techniques including plasma etching. Despite its success, optical lithography is reaching its resolution limits and new structuring techniques are required. Among several candidates, molecular self-assembly and self-organization of structures represent the so-called bottom-up approach. Nanoindentation, thermal scanning probe lithography, local oxidation lithography, dip-pen lithography, extreme UV lithography or X-ray lithography are the leading top-down methods. In this review we focus on another top-down generic technology, namely nanostructuring by charged particle beams used to expose a resist, which can be used as a mask for pattern transfer etching and metal deposition etc.

Nanolithography using charged particle beams can be divided into two approaches: either the particle beam is generated far from the sample and scanned over it using electric or magnetic focusing and deflection optics or the beam is generated at a field-emission tip located in close proximity to the sample and the tip itself is scanned to generate the lithographic pattern. The first approach, termed scanned beam technology, comprises electron and ion beam lithographies and electron/ion beam induced deposition. It has its origins in the scanning electron microscope and, more recently, the scanning ion microscope. Scanning probe lithography, the second approach, also stems from microscopy in the form of scanning tunneling microscopy and atomic force microscopy; the corresponding lithography techniques include scanning tunneling lithography and field-emission scanning probe lithography.

The electron microscope evolved from the use of electron beams from a thermionic cathode in the cathode ray tube pioneered by Braun in 1897 [1], followed by the first practical scanning transmission electron microscope (STEM) built in Berlin by von Ardenne in 1939 [2]. The first commercial SEM was built by Ruska’s team at Siemens in 1939 [3]. Subsequently, Sir Charles Oatley’s team made many scientific and commercial advances at Cambridge University. These included novel secondary electron detectors used for imaging, by Everhart and Thornley [4], and high-intensity electron sources [5] culminating in a series of commercial SEMs [6].

Use of the SEM to write circuit patterns by scanning the beam under computer control to expose a thin layer of electron sensitive polymer resist on the surface of a silicon wafer is a process now universally familiar as electron beam lithography or EBL [7]. EBL has become an important R&D tool for micro and nanoelectronics and for the newer fields of micro and nanoelectromechanics (MEMS/NEMS). The current state of the art in EBL tools is represented by the EBPG5200 system from Raith GmBH which provides a high-intensity beam from a LaB_6_ thermal field emitter (TFE) electron source at energies up to 100 keV and is capable of writing with 8 nm feature resolution over a 200 mm wafer substrate [8]. The state of the art for lithography using scanning proximity probes is the Zyvector system from Zyvex Labs [9]. It provides atomic resolution – the removal of a single atom – using scanning tunneling lithography, but the writing speed is limited to the range of 20–100 nm/s. In general, despite the superior results of charged particle beam lithography for resolution, their relatively low writing speeds and throughput limitations have prevented their use in large-scale manufacturing. Consequently, optical lithography has continued to be the process of choice for integrated circuit manufacture with a range of increasingly sophisticated devices being used to overcome the difficulties of wavelength dependent diffraction limits [10].

However, at the present time, as the critical circuit gate dimension continues to be reduced in pursuit of Moore’s Law [11], and as the 10 nm CMOS gate approaches, charged particle beam tools are becoming increasingly important, not just for R&D and prototyping but also for batch manufacturing. Looking “Beyond CMOS”, single-electron transistors and other quantum devices will become the basic building blocks for the ICs of the future and the ultrahigh resolution and flexibility of EBL and other scanning charged particle tools will, in our view, become increasingly important [12]. In order to compete with optical lithography beyond CMOS, two major properties have to be improved:

1. the lithographic resolution (the size of the smallest structures, which can be fabricated reproducibly)
2. the throughput.

To address these issues, methods such as parallelization by multi-beam or multi-probe systems or the use of other charged particles are being studied. For example, novel charged particle beam tools such as multibeam electron writers [13] are emerging as novel tools of the future. In addition to resist-based lithography, these are capable of writing patterns by electron beam induced deposition [14]. Focused ion beam tools are also becoming increasingly important. The latter include multi-beam ion beam systems employing stencil pattern projection of H^+^ ions (CHARPAN [15] from IMS Austria) and Gaussian single-beam scanning systems using He^+^ ions (ORION NanoFab from Zeiss AG [16]).

In the next sections of this review, we will present the state of the art in scanning beam and scanning probe lithography and discuss methods to improve resolution and throughput.

### 2 Scanning beam lithography

#### 2.1 Scanning ion beam lithography

Scanning ion beam lithography is by no means a new invention, having grown out of electron beam lithography through partnerships between several commercial companies and academic institutions in the 1980s [17]. The systems used were essentially scanning ion microscopes using a liquid metal ion source (LMIS) in place of an electron emitter and electric lenses in place of magnetic coils because of the greater Lorentz force required to deflect the “stiffer” ion beam. Most of these systems used the familiar ^69,71^Ga^+^ LMIS [18]. Such a heavy ion unfortunately produces loss of resist by sputter erosion. Also, because of its size, nuclear stopping in the resist layer limits the penetration depth of the ^69,71^Ga^+^ion beam to a few tens of nanometres at typical beam energies of 20–30 keV. Thus, despite some encouraging early work at Cambridge University and elsewhere [17,19] scanning ion beam lithography (SIBL) was largely ignored for several decades. This was in spite of the higher sensitivity of most resists under ion bombardment and the near absence of the proximity effect that plagues EBL. Backscattered electrons from the resist–substrate interface result in overlap of two features written in close proximity [20]. Ions are over a thousand times heavier than electrons and are consequently not backscattered to the same degree. Mention should also be made of another problem of Ga^+^ lithography which causes a quasi-proximity effect. This is not due to ion backscattering but rather to the shape of the focused ion beam which on the scale of 10 nm or so, exhibits a “halo” in which the central Gaussian spot is surrounded by a second broader lower-intensity region [21–22].

**Figure 1:**
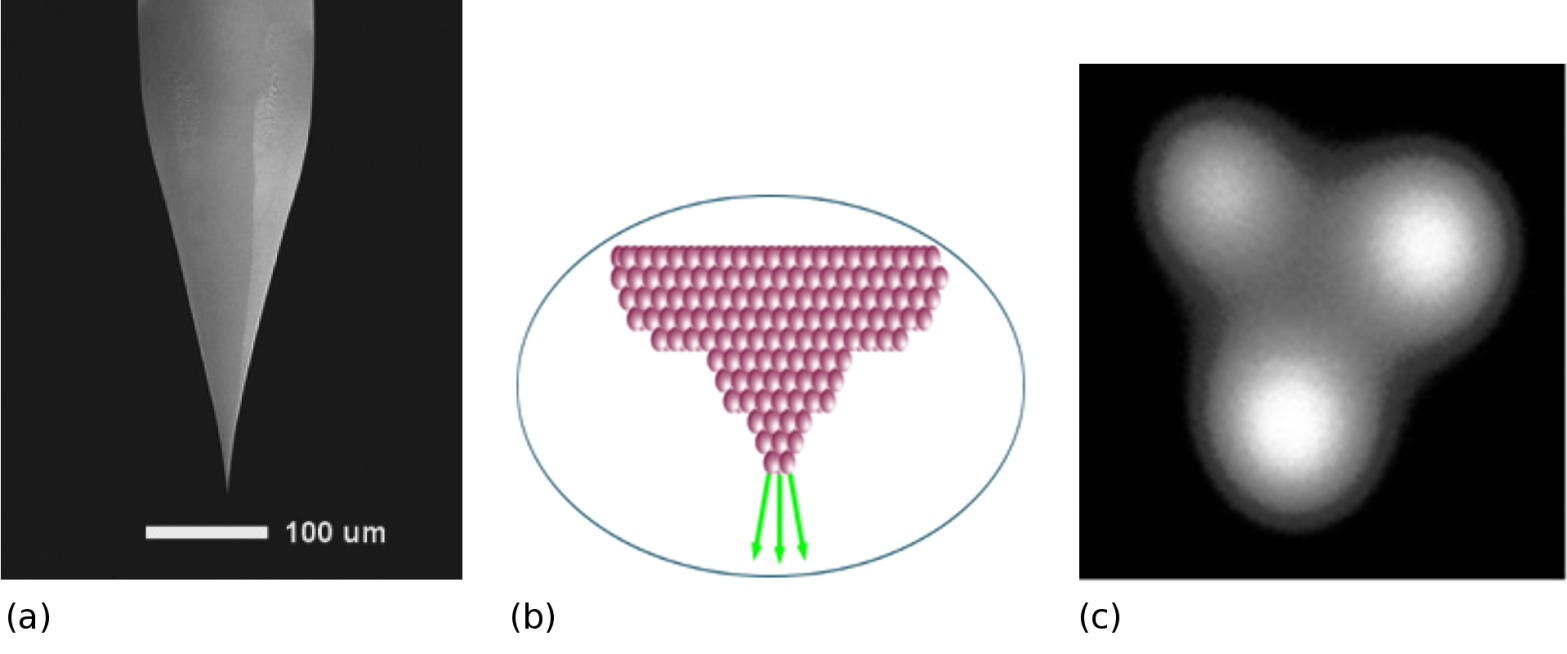
(a) SEM image of ALIS gas field ion source, produced with permission of [24], copyright 2010 Japanese Journal of Applied Physics. (b) Principle of operation of ALIS. (c) W atom trimer emitter at tip providing single atom emitter (ion microscope image obtained by Dr S. A. Boden at Southampton University).

These limitations historically suppressed the adoption of the original Ga^+^ SIBL technology for silicon microelectronics and other applications. There has, however, been a paradigm shift in ion beam microscopy and lithography with the development in 2006 of the atomic level ion source (ALIS) [23]. This can be used to provide a focused beam of the much lighter and less damaging ^4^He^+^ ion. ALIS is a highly developed version of the gas field ion source (GFIS), the operation of which is shown schematically in Figure 1 [23–24].

The non-uniform high electric field at the atomically sharp tip of a tungsten needle maintained at cryogenic temperature (below −150 °C) causes gas atoms to be polarized and driven along the surface of the needle to its tip where they are field-ionized [25]. The effective ion source dimensions are therefore on the atomic scale since emission occurs from a single W atom; the resulting source diameter is approximately ten times smaller than for the Ga^+^ LMIS which has an ultimate source size of 3 nm [26]. The ALIS source consequently has an extremely high brightness estimated to be approximately 5 × 10^9^ A·cm^−2^·sr^−1^ at an ion energy of ca. 30 keV.

**Figure 2:**
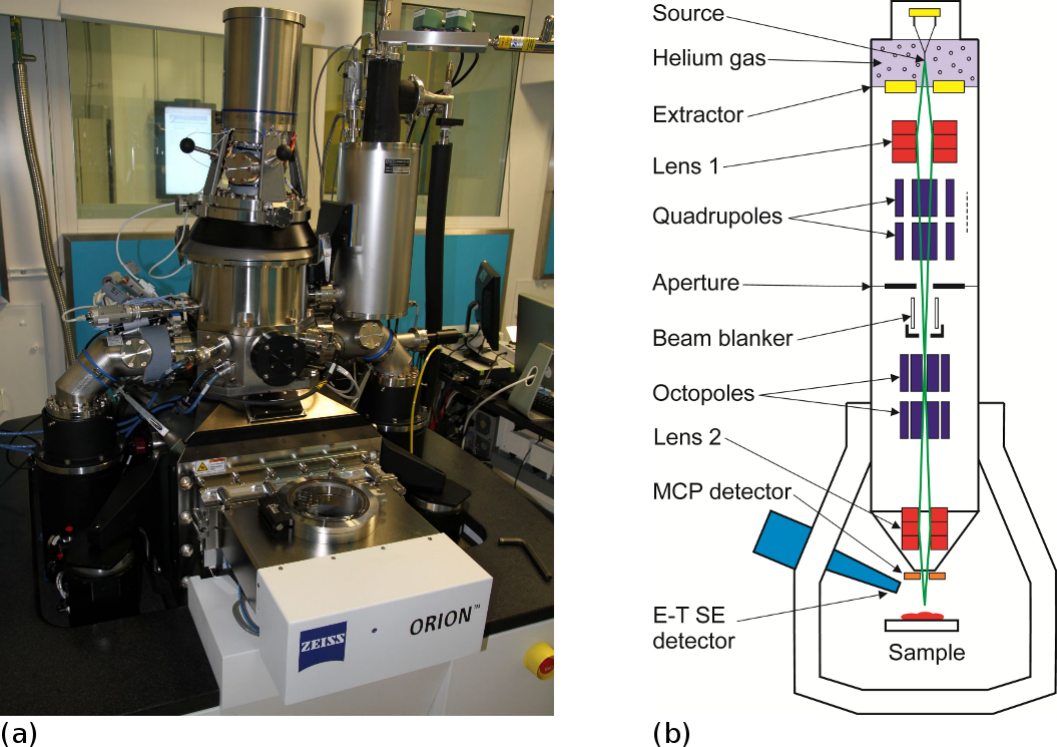
(a) ORION Plus He Ion Microscope from Zeiss AG, located at University of Southampton. (b) Schematic of He^+^ ion focusing column.

The ALIS source has been incorporated into the ORION scanning ion microscope system, which is similar to the previous Ga^+^ microscopes in most other respects, by Zeiss AG. The system shown in Figure 2 is the ORION Plus helium ion microscope [16]. The ultimate edge resolution of this tool in the range of beam energies 20–30 keV is better than 0.5 nm. This, together with the minimal He^+^ ion damage effects compared with the earlier Ga^+^ systems, and reduced proximity effect, are the reasons for the renewed interest in ion beam lithography in the form of scanning helium ion beam lithography or “SHIBL”. The ultimate resolution is determined by a combination of ion beam diameter and resist properties with subsequent ion scattering and ionization cascade. It is not due to de Broglie quantum mechanical wavelength of the charged particle as seen through the comparisons in Table 1.

**Table 1:** Comparison of quantum mechanical wavelengths for ions and electrons.

beam energy [eV]	electron wavelength [nm]	Ga ion wavelength [nm]	He ion wavelength [nm]

100	0.12	0.00034	0.006
1000	0.04	0.001	0.002
10,000	0.012	0.000034	0.0006
100,000	0.004	0.00001	0.00002

**Figure 3:**
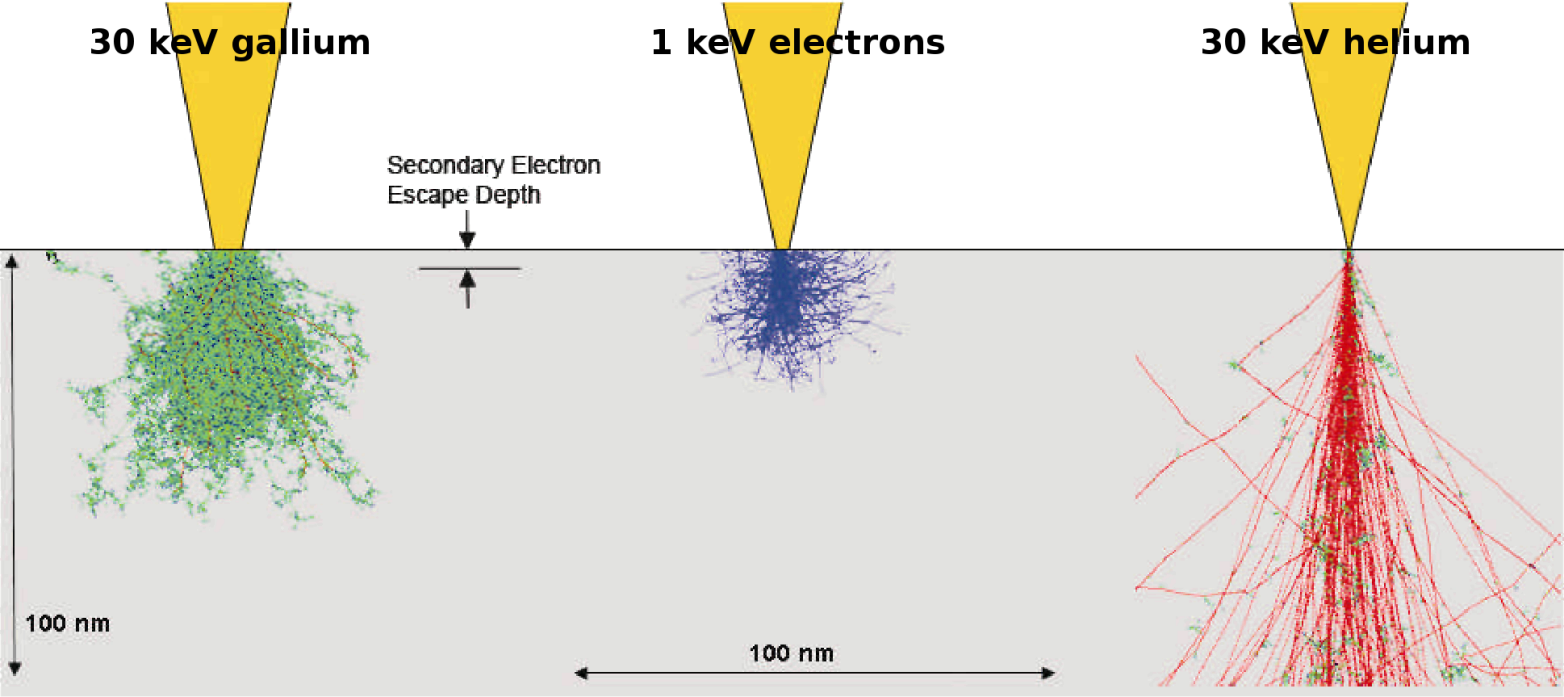
Electron and ion beam substrate penetration – Monte Carlo simulations of charged particle paths showing forward and back scattering. Reproduced with permission through Creative Commons Attribution (CC BY) from [30], 2007 AIP Conference Series.

These wavelengths determine the diffraction contribution *d*_df_ to the ultimate probe size for all forms of charged particle microscopy and lithography, according to the RPS formula [27–28],

[1]
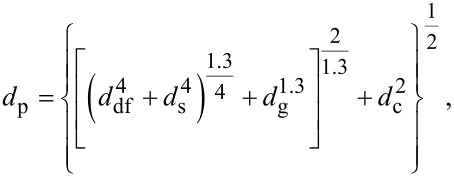


where *d*_s_ and *d*_c_ are the contributions to the final spot size due to spherical aberration and chromatic aberration. The Gaussian optical image size magnified through the lens optics is *d*_g_. The quantum mechanical wavelength contribution can be neglected for ions (see Table 1). In the critical regime dominated by chromatic aberration, the energy spread of He^+^ ions from the ALIS is in the range 0.25–0.5 eV FWHM, which is one order of magnitude less than for Ga^+^ ions from the LMIS [29–30]. For 100 eV electrons the wavelength is more than 10% of the target 1 nm resolution. This is of particular relevance to lithography using electrons generated in proximity probe (STM) systems and will be discussed further in Section 3. After the ultimate probe size is determined, the ability to perform lithography using charged particle beams and the resolution obtained is set by the scattering of the beam in the resist layer and the underlying substrate. Scattering and range depend upon a combination of electron scattering and nuclear scattering. The latter is of particular importance for heavy ions like Ga^+^ and causes damage to the resist through sputtering and loss of substrate crystallinity. The situation for Ga^+^ ions is further complicated by the property of Ga^+^ as a p-type dopant of silicon. Scattering and range can be calculated using Monte Carlo simulation codes with typical results as shown in Figure 3 [30]. The advantages of SHIBL using He ions over SIBL using Ga ions is clear from the reduced lateral spread of the beam in the top 10–20 nm of the surface where the ultrathin resist layer is located for nanolithography. In practice, the effect of the resist can be ignored and the lateral spread is determined by the wafer substrate alone. The resist layer is exposed not by the primary ions but by the electrons they generate, which have lower energies compatible with breaking the chemical bonds of the resist – the so-called “δ-rays”. The main points of comparison are summarized in Table 2.

**Table 2:** Comparison of beam diameters and scattering effects for different charged particle lithography methods.

method	Ga^+^ SIBL	EBL	He^+^ SHIBL

probe size	>1 nm	>1 nm	<0.5 nm
scattering effects	no proximity effect	proximity effect	low proximity effect

**Figure 4:**
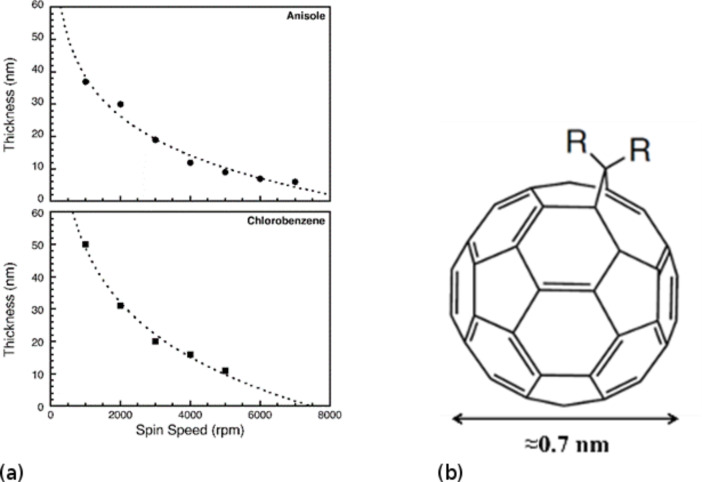
(a) Spin-coating results for fullerene resists HM-01A and HM-01C using anisole and chlorobenzene solvents, respectively. (b) Schematic of the monoadduct methanofullerene molecule used in the HM resist series [40].

Before the EU Framework 7 project “Single Nanometre Manufacturing” (SNM) [12], the state of the art in SHIBL was represented by work at TU Delft and Hewlett-Packard Labs [31–32] which demonstrated He^+^ lithography with linewidths down to 4 nm in hydroxyl silsesquioxane (HSQ) negative tone electron beam resist. Following this, the SNM project has explored the use of a number of new resists chosen for their superior performance in plasma etch pattern transfer applications. These were required since HSQ has poor etch resistance in its as-developed state and it is necessary to process it further using an electron post-development curing process in order to render it compatible with fluorine etch and other plasma chemistries [33]. These new resists include novel fullerene formulations from Irresistible Materials Ltd (IM) and Birmingham University, UK, which have demonstrated etch selectivity with respect to Si of 9:1 in fluorine plasma chemistry [34]. These resists are polymerized from the C_60_ molecule, selected for its sub-nanometre dimensions (0.7 nm) and its stability [35]. Resist screening experiments on one of these resists with the experimental codename HM-01 have revealed exceptional plasma etch resistance and stability [36] and this was chosen for scanning He^+^ ion beam lithography (SHIBL) experiments. In order to enable sub-10 nm patterning, an ultra-thin resist film, with small molecules is required. High-fidelity pattern transfer via plasma etching requires high carbon content in the resist, with as many of the carbons in a ring structure as possible, as is the case of a monoadduct methanofullerene derivative. The fullerene molecule gives the maximum possible value of ring parameter and the minimum possible Ohnishi number [37]. The sub-nanometre fullerene molecule forms amorphous films and has been shown previously to be suitable for electron beam lithography [38]. Derivatisation of the fullerene renders it soluble in common spin-coating solvents. A number of different fullerene derivatives have been spin-coated and patterned using electron beam lithography [38–39]. An ultrathin-film, ultrahigh-resolution resist for He^+^ ion beam exposure with high overall carbon content and good solubility is now available from IM Ltd. The structural class of the IM fullerene derivative is indicated in Figure 4 [40]. The carbon content of the material is ca. 95 wt %; the Ohnishi parameter is ca. 1.26, and the ring parameter is ca. 0.87. The resist can be formulated in various solvents, including chloroform, chlorobenzene and anisole. Chloroform was rejected as a solvent for SHIBL patterns due to poor results seen in EBL patterning and due to its hazardous nature, and development has been focused on two formulations: HM-01A using anisole solvent and HM-01C using chlorobenzene. The spin-coatability of the two formulations is shown in Figure 4a.

**Figure 5:**
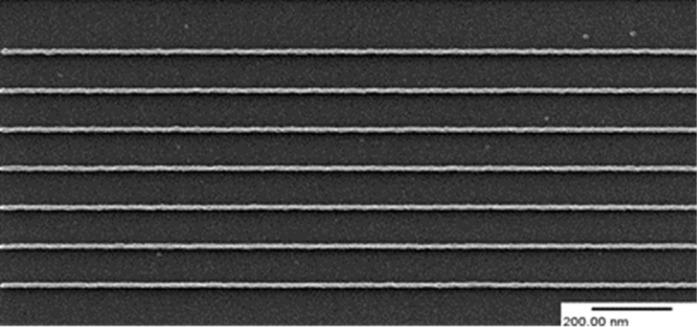
Isolated dose-optimised SHIBL experiments on HM01 fullerene resist: 8 nm wide sparse exposed using 30 keV He^+^ ions [41].

Figure 5 shows 8 nm isolated lines written with a line dose of 0.08 nC·cm^−1^ in a 10 nm layer of HM-01A negative tone fullerene resist [41]. (Many novel resists require physical vapour deposition but HM-01A and HM-01C have the advantage of being spin-coatable.)

**Figure 6:**
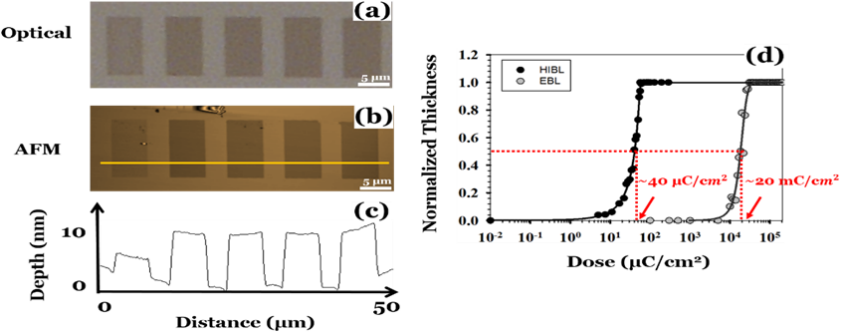
Results of SHIBL at 30 keV beam energy: (a) optical micrograph, (b) AFM image, (c) line profile (HM-01 fullerene resist thickness ca. 10 nm). He^+^ doses left to right: 37.9, 56.5, 85.0, 128.0, 192.0 μC·cm^−2^, (d) Comparison of the dose response curves for HM-01 in SHIBL and EBL. Sensitivities are 40 µC·cm^−2^ and 20 mC·cm^−2^, respectively, revealing a 500-fold increased sensitivity in SHIBL [41].

A comparison of the sensitivity of the HM resists for He^+^ ion beam lithography at a beam energy of 30 keV and that for electron beam lithography at the same beam energy is shown in Figure 6 [41]. The HM resists are up to 500-times more sensitive in SHIBL than in EBL at the same beam energy. This is markedly better than HSQ for which the corresponding figure is 4.4-times. The contrast in SHIBL is higher for the anisole version HM-01A.

**Figure 7:**
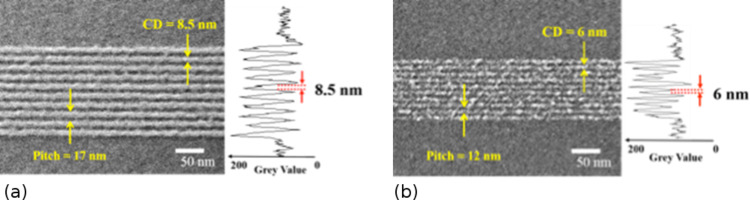
HIM image of dense (1:1) single-pixel features exposed at (a) 0.09 nC·cm^−1^ and (b) at 0.04 nC·cm^−1^ in ca. 10 nm thick HM-01A fullerene resist, with line scans of secondary electron intensity providing measurements of linewidth [41].

The results of SHIBL in 10 nm thick HM-01A fullerene resist at 30 keV He^+^ ion beam energy are shown in Figure 7 for dense features [41]. Figure 7a shows dense single-pixel features exposed at a line dose of 0.09 nC·cm^−1^. The SE contrast measurement reveals continuous lines with 8.5 nm line width and 17 nm pitch. Figure 7b shows that 6 nm lines on 12 nm pitch were resolvable but the lines were broken at a line dose of 0.04 nC·cm^−1^, which is equivalent to 25 ions per nanometre, i.e., a signal-to-noise ratio of 5:1. The line discontinuity is caused by shot noise producing missing pixels. Thus 6 nm represents the current limit of 1:1 dense features using SHIBL to expose HM-01A fullerene resist.

**Figure 8:**
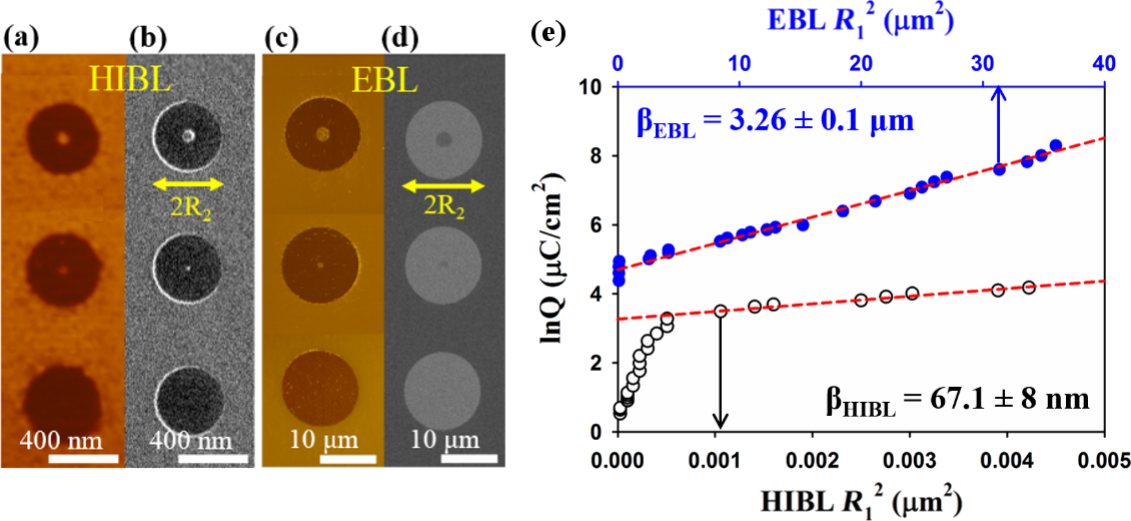
(a) AFM and (b) corresponding HIM, (c) AFM and (d) corresponding SEM images of doughnuts fabricated using SHIBL and EBL with fixed outer radii (*R*_2_) of 200 nm and 7 μm, respectively, and varied inner radii (*R*_1_). (e) Comparison of the proximity effect for SHIBL and EBL on 20 nm thick PMMA [42].

As discussed earlier, the proximity effect limits the resolution and fidelity of high-density sub-10 nm patterns. Sidorkin and co-workers [31] showed that SHIBL does not suffer from a significant proximity effect. This assertion has been verified in SHIBL experiments by Shi et al. [42] using PMMA resist, the positive tone resist for which the classic EBL proximity effect experiments were conducted [20]. The method follows that employed for EBL by Stevens et al. [43] and by Boere et al. [44], and involves exposing doughnut-shaped areas with various inner radii, *R*_1_, and determining the dose required to fully clear their centres (through the proximity effect). In Shi’s work, the PMMA was spun to a thickness of 20 nm and prebaked for 70 s at 180 °C; development was carried out in MIBK/IPA (1:3) for 60 s. For SHIBL, the outer radius of the doughnut was fixed at *R*_2_ = 200 nm while the inner radius was varied in the range from 5 to 125 nm. The dose was varied in the range of 0.3–200 µC·cm^−2^. The SHIBL beam conditions were 30 keV He^+^ energy with an ion current of 0.3 pA. For comparison to EBL, the same resist was exposed using a 30 keV electron beam in a SEM with *R*_2_ fixed at 7 µm, *R*_1_ varied in the range of 0.04–6.5 µm and the dose varied in the range of 80–4000 µC·cm^−2^. Images showing examples of resulting doughnut structures are presented in Figure 8a–d.

Equation 2 shows the relationship between the exposure dose and the forward scattered and “backscattered” components [44]:

[2]
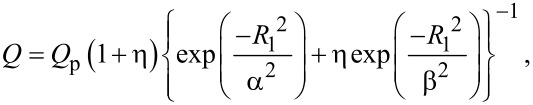


with *Q* being the exposure dose required to clear out the resist from the centre of the doughnut due to the proximity effect, *Q*_p_ being the threshold dose required for full clearance of the resist, *R*_1_ being the inner radius of the doughnut, α being the standard deviation of the forward-scattered deposited energy distribution, β being the standard deviation of the “backscattered” energy distribution and η being the ratio of forward scattered energy deposited to “backscattered” energy deposition in the resist.

For values of *R*_1_ >> α, Equation 2 may be approximated to a linear relationship between ln *Q* and *R*_1_^2^ (Equation 3):

[3]
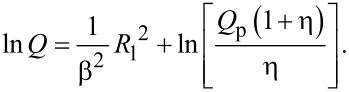


Equation 3 was fitted to the experimental results as shown in Figure 8e, giving a standard deviation of β = 67 nm for the spatial distribution of the backscattered energy for SHIBL, which is almost 50-times smaller than the corresponding EBL value. Thus, while there is some residual proximity effect in the case of SHIBL because of the low mass of the He^+^ ions, these effects are negligible when compared to those due to electron backscattering in EBL. Future work should seek to establish the origins of the proximity effect in SHIBL, i.e., whether it is due to secondary electrons or true ion backscattering. This will be revealed by further experiments using substrates of varying atomic mass. Gold should produce a greater proximity effect than aluminium if ion backscattering dominates.

#### 2.2 Electron beam lithography: recent developments

**Figure 9:**
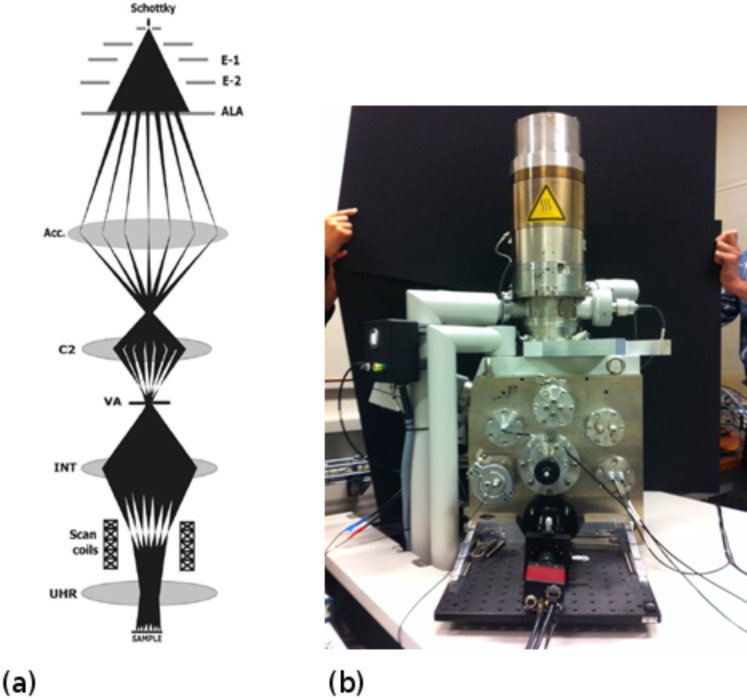
(a) Schematic of the multibeam EBL system at TU Delft [45–46]. (b) Experimental multibeam tool using a Nova Nano SEM from FEI Co as platform.

**2.2.1 High-throughput/high-resolution EBL.** As described in the Introduction, electron beam lithography (EBL) has a long history as the principal charged particle method for sub-micrometre lithography. As with all charged particle techniques to date, EBL normally uses a single charged particle beam. The SNM project, in contrast, included a substantial programme of work at TU Delft to develop a novel multibeam EBL system (see Figure 9). This has several advantages over a single Gaussian beam tool, including higher throughput, as each of the multiple beams can be used to write a separate chip in parallel, overcoming the slow serial nature of conventional single-beam EBL. Even more novel is the capability of multibeam EBL systems to write different chips in a single manufacturing step, whereby each of the chips written in parallel by the multiple beams may have different individual device and circuit layouts. This will be a major advantage in R&D and batch manufacturing, enabling multiproject wafers for chip-development purposes.

**Figure 10:**
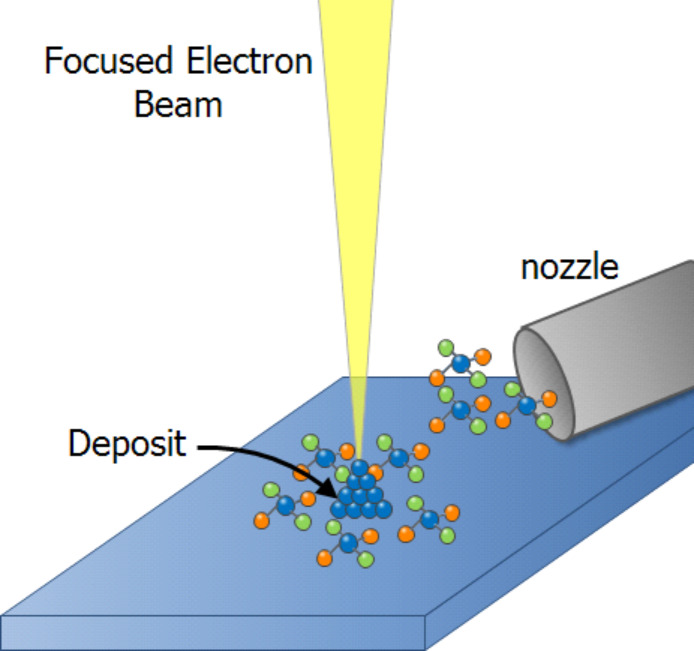
Schematic of electron beam induced deposition (EBID).

The prototype multibeam tool at TU Delft has 196 electron beams and has been used as an electron beam deposition (EBID) writer. Negative tone pattern generation is achieved by electron beam decomposition of a gas precursor as shown schematically in Figure 10.

EBID is a technique with a potentially higher spatial resolution than conventional resist-based EBL. The next section briefly reviews EBID and its counterpart electron beam induced etching (EBIE). The generic term for both techniques is focused electron beam induced processing (FEBIP).

**2.2.2 Focused electron beam induced processing.** Focused electron beam induced processing (FEBIP) is a high-resolution direct-write nanopatterning method comprising two complementary techniques, namely electron beam induced deposition (EBID) and etching (EBIE). The advantages of FEBIP lie not only in the high resolution achievable by the use of focused electron beams but also in their inherent ease of use and flexibility when compared to conventional lithographic techniques. A variety of materials can be deposited or etched by the use of different precursors [47] and the method requires no resist or sample preparation. In addition, there are no restrictions on the substrate to be patterned, accommodating everything from flat wafers to AFM tips. Extensive reviews of EBID and EBIE can be found in [47–50]. Due to the versatility of FEBIP, it has been used for several different applications such as the fabrication of electrodes, etch masks, nanorods, 3-dimensional, plasmonic and superconducting nanostructures [51–52] not all of which require the highest achievable resolution. In this section we present a brief review of sub-10 nm FEBIP, focusing on the possibilities for patterning offered by this technique that are not easily achievable by any other, as well as on the ultimate resolution achievable. We also give a few examples of ultrahigh-resolution work performed using STM-based EBID and pattern transfer. As described above, EBID can be carried out in an electron microscope by focusing the primary electron beam on the substrate in the presence of adsorbed precursor gas molecules delivered from a nozzle close to the sample surface. The electrons interact with the substrate generating high-energy backscattered and low-energy (<50 eV) secondary electrons all of which interact with the molecules causing them to dissociate. The non-volatile dissociation fragments form a deposit on the substrate and in this manner patterning can be carried out by scanning the beam. EBIE is essentially similar with the exception of some additional processes involving the etch products.

**Figure 11:**
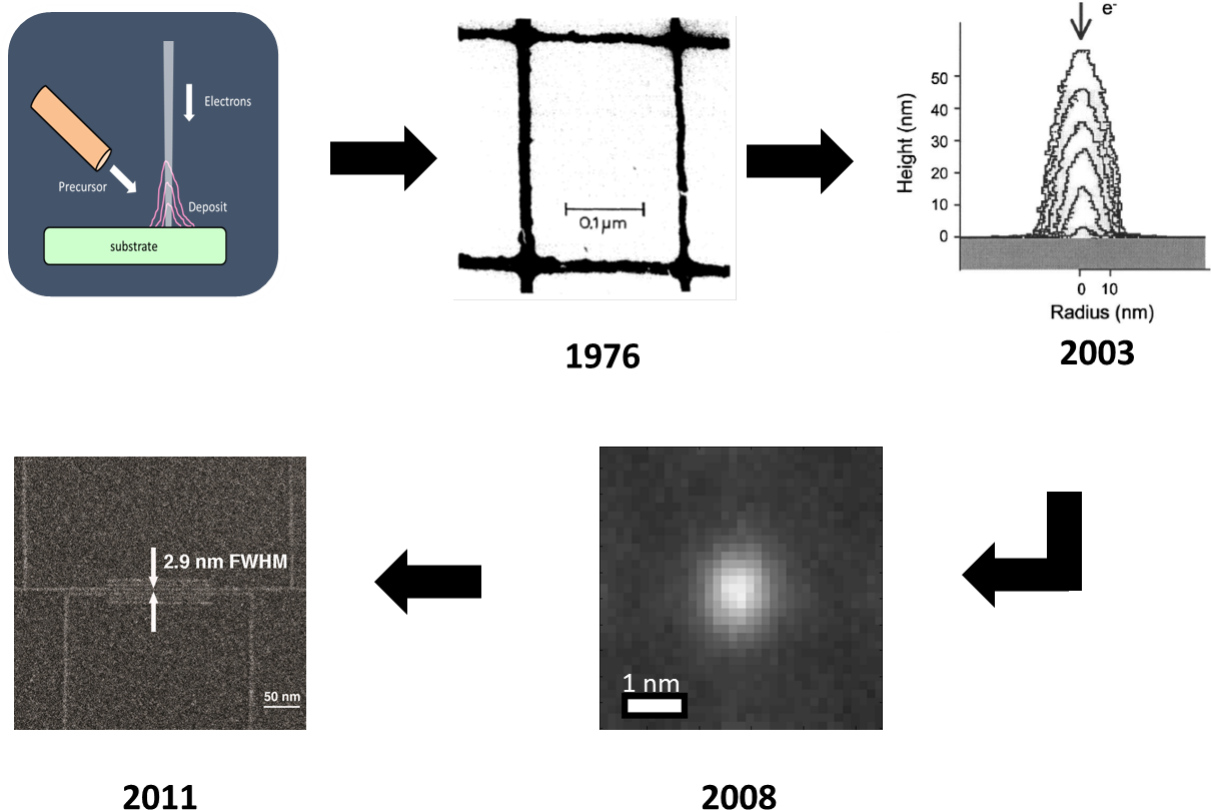
Schematic representation of the evolution of high-resolution electron beam induced deposition as a nanopatterning technique since its first demonstration in 1976. (Clockwise order) Schematic of EBID, 8 nm lines patterned by Broers et al. on a thin membrane [55]; simulation of dot grown on a thin membrane using a zero-diameter electron beam demonstrating the relevance of deposit-generated SEs in EBID [70]; experimental demonstration of dots with average diameter of ca. 1 nm patterned on a thin membrane by EBID [64]; direct patterning of 3 nm dense lines on bulk Si/SiO_2_ by EBID [65]. Reprinted with permission from [55] and [70], copyright 2003, 2013 AIP Publishing; reprinted with permission from [65], copyright 2011 American Vacuum Society.

**2.2.2.1 Electron beam induced deposition.** We begin with a brief review of EBID, which addresses the fabrication of dots and lines in SEM, TEM and STM on bulk and thin film substrates, as well as sub-10 nm FEBIP for specialised applications. Since the darkening due to decomposition of surface contaminants was first observed while imaging in the SEM, this process was exploited to deposit insulating thin films [53] and sub-micrometre patterns [54] of a range of materials in a controlled manner by scanning the electron beam. The high resolution of this technique was demonstrated as early as 1976 by Broers et al. [55] who patterned EBID lines from hydrocarbon contamination in the SEM chamber, using it as a mask to etch 8 nm wide metallic gold palladium lines into a carbon film. Silvis-Cividjian et al. [56] and van Dorp et al. [57] achieved still smaller lines (<5 nm) by EBID on a thin membrane. The development of EBID for reliable nanofabrication has required several issues to be addressed, such as different kinds of proximity effects [58–59] leading to deposit broadening while patterning dense lines and dots, variation of deposit morphology as a function of the beam current and precursor flux [60], and the role of secondary electrons in the deposition process. The use of EBID for direct deposition of sub-5 nm dots on a membrane was demonstrated by Tanaka [61] and Mitsuishi [62] in a TEM and by van Kouwen [63] in an SEM where the difficulty in imaging structures near the resolution limit is also highlighted. Van Dorp et al. [64] studied in detail the initial stages of EBID, which are critical for patterning ultrasmall structures, by growing dots as small as 0.72 nm (FWHM) in a TEM. They determined that the process was limited by the statistics governing the dissociation process. Precursor diffusion over the sample surface is also known to be relevant [65–69], except when working in the electron current limited regime, demonstrating the significance of patterning strategy. The first demonstration of sub-5 nm dense lines deposited directly by EBID on a bulk substrate in an SEM was by van Oven et al. [65] using the organometallic precursor trimethyl(methylcyclopentadienyl)platinum(IV) (MeCpPtMe_3_). A combination of low beam current, low working distance and multiple pass patterning, synchronised with the 50 Hz disturbances, resulted in the successful fabrication of 3 nm Pt/C lines and spaces on bulk silicon. Figure 11 shows schematically the evolution of EBID into a controlled nanopatterning technique since the first demonstration of its high resolution, followed by simulations and experiments on thin films to study the ultimate resolution achievable.

This has ultimately resulted in the successful fabrication of dense lines on a bulk substrate, demonstrating the evolution of EBID into a well-controlled nanopatterning technique and a good candidate for lithography. One of the major considerations in this application is the purity of the deposited material. This has been reported in detail in the literature, with the demonstration of both post processing and in situ techniques for achieving, inter alia, high-purity metallic deposits [71–73] and will not be discussed further here. Another important issue is throughput, which although lower than that of optical or EUV lithography, because of the sequential exposure, can be overcome by using a multibeam SEM [14] for high-speed parallel patterning, as already mentioned in Section 2.2.1. However, we limit the discussion here to the question of the maximum resolution attainable using EBID. Some analytical and numerical calculations have been carried out with the aim of providing quantitative results. Utke [67] studied the relation between electron flux, precursor dissociation, depletion and diffusion, deriving scaling laws that allow for the determination of EBID resolution as a function of the balance between these parameters. Silvis-Cividjian [70] developed a Monte Carlo model for EBID, specifically for high-resolution deposits, taking into account the energy and spatial distribution of SEs. This elucidates the crucial role played by the SEs generated in the deposit itself in determining the final deposit size, contrary to the classical model, developed for SEM imaging, which is based purely on scattering in the substrate [74]. This was further studied by van Dorp et al. [75–76] who performed experiments on very thin membranes with different precursor fluxes to verify the role of SEs in EBID. This work demonstrates the need for better modelling of the SE emission profile, as also carried out by Fowlkes et al. [77] and Smith et al. [78] to enable the prediction of 3D deposit geometry and growth rate as a function of the patterning parameters. The high resolution of the technique has been utilised in the fabrication of isolated structures, for example by Koops et al. [79], and by Frabboni et al. [80] who attempted to overcome the resolution limit due to the presence of a bulk or thin substrate by growing suspended nanowires by EBID on the tip of a tilted pillar, achieving a lateral resolution of 5 nm. Sub-10 nm gaps have been fabricated in devices in [55,81–83] using EBID directly, as a mask or in combination with a metallic layer to enable specific functionality. EBID has been used for several novel applications such as the deposition of magnetic nanostructures by Pai [84] and Kent [85] using STM combined with CVD. As another example, 5 nm GaN quantum dots were deposited by Crozier [86] by EBID from a specially tailored precursor resulting in high-quality uniform deposits on a thin film of Si/SiO_2_. Shimojo [87] demonstrated the deposition of self-standing nanorods, 10 nm in diameter, by electrons in the presence of a chloride-containing precursor. Remarkably, the nanorods do not contain the precursor material but are instead formed from the substrate material.

**2.2.2.2 Electron beam induced etching.** Focused electron beam induced etching is another direct-write technique used for high-resolution nanopatterning. Adsorbed precursor molecules are dissociated by the electron beam, leading to the formation of reactive fragments that react with the substrate to locally volatilize it. Although analogous to gas-assisted etching by ions, it is in fact a chemically selective technique, complementary to EBID in that it is top-down, with a significant advantage over ion milling due to the absence of sputtering. It therefore has wide applications, including use on samples that cannot withstand ion exposure, e.g., due to damage susceptibility. The first report of EBIE to our knowledge was in 1979 [88] when SiO_2_ and Si_3_N_4_ substrates were etched by electrons in the presence of XeF_2_ gas, whereas no etching was observed in the presence of either electrons or gas molecules alone.

Since then, several reports and applications of gas-assisted etching using focused electron beams have appeared in the literature including EUV mask repair [89], cutting of nanotubes [90] and etching of holes in thin films. In this review we enumerate the major contributions in high-resolution EBIE which, although relatively few in number, clearly demonstrate the potential of the technique. The fabrication of nanopores, for example, is interesting for the localisation and analysis of biomolecules. Miyazoe [91] used a conventional SEM fitted with an external reservoir for precursor injection to etch holes as small as 18 nm in diameter in a thin carbon membrane using both H_2_O and XeF_2_. Yemini [92] fabricated a nanopore array, demonstrating control over the feature size by etching a series of holes with diameters of 17–200 nm in a Si_3_N_4_ membrane using XeF_2_. From the point of view of high-resolution lithography, an interesting result was reported by Ganczarczyk [93] who etched 30 nm lines into a GaAs substrate in an SEM, using XeF_2_ as the precursor. At such high resolution, the line edge roughness (LER) is an important parameter. While it is reported as being considerably smaller than the line width, it would be of interest to study it further to determine the origin of LER and its dependence on experimental parameters and substrate properties. Due to the time taken for etching, drift in the system is also noted as being significant. This is another factor requiring optimisation since it affects the quality of etched lines.

**Figure 12:**
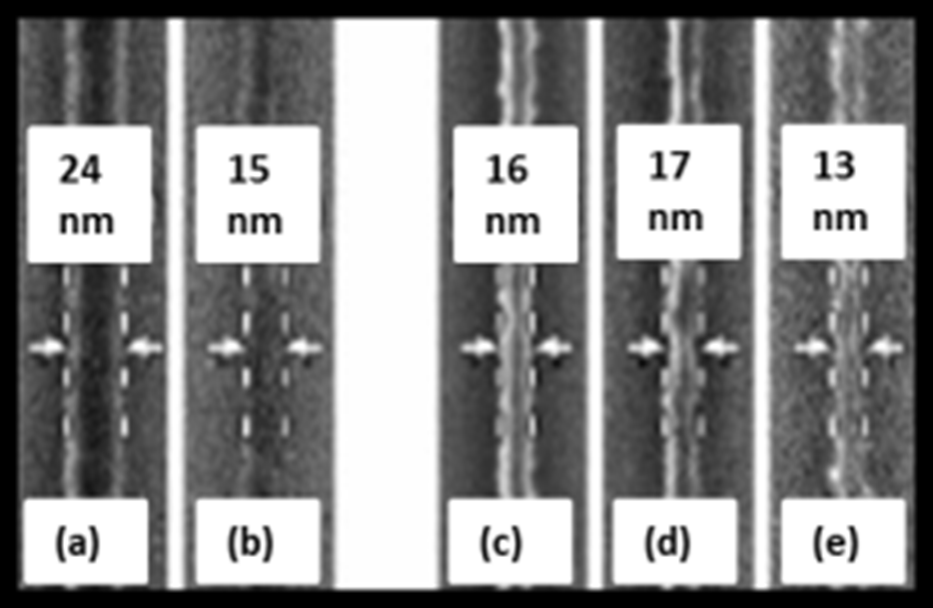
Carbonaceous nanowires on bulk SiO_2_ such as the one in (a) slimmed by electron beam induced etching in the presence of water in an ESEM resulting in wires of different widths (b–e). Adapted with permission from [94], copyright 2007 American Chemical Society.

Understanding the chemical kinetics of EBIE is important for achieving greater control over the technique and to determine the ultimate process resolution. In one of their early papers, Toth and Lobo [94] suggested that the etching was driven by SEs generated at the surface of the deposit or the substrate. Based on this, they demonstrated the fabrication of 4–7 nm wide gaps in a carbonaceous nanowire by an unconventional method. Using a stationary beam, pits were first etched at the desired location by EBIE with H_2_O in an environmental scanning electron microscope (ESEM). The field of view containing the nanowire was then scanned repeatedly in the presence of the precursor until a gap was created due to the increased SE yield from the nanowire sidewalls. The enhanced contrast of the nanowire edges seen in the SE image lends support to their theory. The advantage of working in an ESEM was the possibility of charge counteraction, allowing the use of insulating substrates like SiO_2_ that are common in electrical measurements. Another important application of EBIE demonstrated there was the modification of as-deposited EBID structures to alter their physical or chemical properties. Carbonaceous nanowires of about 24 nm width deposited by EBID were slimmed by repeatedly scanning the field of view containing the deposits in the presence of water. In this manner, nanowires with widths down to 13 nm were achieved, as shown in Figure 12.

The specific case of EBIE of SiO_2_ using XeF_2_ was studied by Randolph et al. [95] who performed experiments to study the etch rate as a function of beam parameters and proposed a two-step mechanism governing the process. A more general continuum model incorporating the electron profile, precursor adsorption, diffusion and secondary reactions involving the etch product is presented in [96] to explain experimentally observed etch profiles in terms of the operating conditions describing electron-current-limited and precursor-limited regimes. Although conventional EBIE models assume the etching rate to scale linearly with the rate of dissociation of the precursor, Martin et al. [97] described another important effect, viz., surface-site activation due to exposure to the electron beam resulting in the formation of surface defects. They performed EBIE of ultra nanocrystalline diamond as well as numerical simulations to study this effect and demonstrated that the rate is limited by the number of active sites available for etching.

In the field of sub-10 nm EBIE, considerable work needs to be done both experimentally and theoretically. The resolution in EBIE, as in EBID, is usually limited by the distribution of electrons (PEs, SEs and BSEs) at the sample surface. Using simulations, Lobo et al. [98] presented simultaneous EBIE and EBID within the beam profile as a technique to overcome this limit on both thin film and bulk substrates. It is well known that, during EBIE, the competing deposition due to EBID from hydrocarbons in the chamber influences the resolution as well as the etch rate. Upon changing the electron flux, an abrupt transition between EBIE and EBID was reported in [99]. Making use of the difference in flux as well as in dissociation cross sections of the two precursor gases, it was predicted that the electron current can be tuned to achieve sub-beam sized radially symmetric deposits by simultaneous EBIE and EBID. From this simulation, using a 4 nm diameter electron probe, ring-shaped deposits smaller than the electron beam diameter were shown to be possible. However, quantitative models to predict the maximum resolution attainable as a function of experimental parameters would be essential for making EBIE a viable nanopatterning technique.

**Figure 13:**
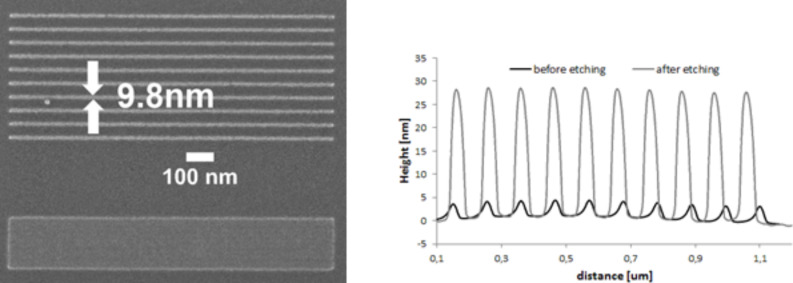
Left: SEM micrograph of an EBID mask consisting of 17 nm lines at 50 nm spacing, transferred into the silicon substrate using fluorine etch. Right: AFM profile showing a height ratio before and after etching of 8 [105].

**2.2.2.3 EBID and large-area applications**. From the previous section it is clear that EBID is a proven high-resolution direct-write lithography technique. However, for large area manufacturing, as targeted in the SNM project, one would prefer better control over the composition of the deposits, and high throughput. The first is an issue that is still being investigated by several groups and the latter can be achieved by using multibeam systems. To circumvent the composition issues of EBID one can use the deposits as a mask for a subsequent pattern transfer into an underlying substrate. The multibeam scanning electron microscope (MBSEM) introduced in Section 2.2.1 can be used to enhance the throughput by a factor of 196 [14,45–46]. But, for large-area manufacturing of single nanometer structures and devices, massively parallel lithography techniques, such as nanoimprint lithography (NIL), are preferred. In NIL, nanostructures are fabricated through mechanical deformation of a soft polymer by pressing a mold (or stamp) into it [100]. Typically, high-resolution NIL stamps are made by fabrication of patterns on top of a silicon substrate using EBL, or by transferring these patterns into the underlying silicon substrate, to increase the aspect ratio [101]. For high-resolution stamps, EBID would be preferred over EBL. The pattern transfer of sub-100 nm EBID patterns, even down to 20 nm has been reported in the literature [81,102] as well as applications for device fabrication [103–104]. However, pattern transfer of sub-20 nm EBID structures is far more difficult, and has only recently been reported by Scotuzzi et al. [105]. These authors propose to use EBID to fabricate stamps for sub-10 nm NIL, followed by a pattern transfer step using plasma etching to increase the aspect ratio of what are usually very shallow structures. They carried out preliminary experiments to gain understanding with sub-10 nm EBID masks, using hydrogen bromide (HBr), chlorine (Cl_2_), chlorine and boron trichloride (BCl_3_/Cl_2_) and fluorine chemistries (SF_6_/C_4_F_8_). The quality of the etching process is mostly determined by the directionality of the etching, which is preferably anisotropic, and the surface quality after etching. The height ratio, defined as the ratio between the height of the mask before etching and the height of the structure after etching, allows an estimate of the relative etching rates of the substrate materials to be made and hence the selectivity. A set of EBID masks with structures between 8 and 20 nm were fabricated using the platinum precursor MeCpPtMe_3_ on a silicon substrate with a 20 kV, 40 pA beam providing a dose of 4000 C·m^−2^ in ultrahigh-resolution (UHR) mode. The patterning as well as the inspection of the masks before and after etching was done in an FEI Nova Nano Lab 650 SEM. The lateral dimension of the structures was obtained from the SEM images, while the feature height was calculated from the profile of a Bruker Nanoscope V atomic force microscope (AFM). The best results were obtained with the fluorine chemistry that is known to be isotropic, but the presence of C_4_F_8_ allows the formation of a sidewall passivation layer that improves the directionality of the etching. Figure 13 shows the SEM micrograph of a mask with 17 nm wide lines at 50 nm spacing, etched into silicon, resulting in a height ratio of 8, as taken from the AFM profiles in Figure 13, and lines of 9.8 nm width.

The combination of high-resolution EBID patterning using multiple electron beams and the pattern transfer into the underlying stamp material allows for the fabrication of high-resolution NIL stamps. Replicating such master stamps in a step-and-repeat NIL process will result in large-area NIL stamps that can be used for high-throughput, high-resolution and large-area applications.

### 3 Scanning probe lithography

Scanning probe nanolithography has its origins in the microscopy techniques of atomic force microscopy (AFM) and scanning tunneling microscopy (STM). In both cases, a probe is scanned over a sample and the interaction is used to study the sample properties. For AFM, the atomic force between a sharp tip at the end of a cantilever beam and the sample surface is measured by read-out of the cantilever bending. The STM uses the tunneling current between a tip and the surface to obtain information about the sample surface [106–107]. Scanning probe nanolithography uses the interaction of such a tip with the sample to nanostructure its surface. For the purpose of this review*,* scanning probe nanolithography (SPL) refers to the application of these proximity probes for nanolithography of polymeric resists and we therefore focus on electron-based SPL methods. However, we will mention briefly the diversity of SPL methods that take advantage of the multiplicity of probe–sample interaction mechanisms [108–112]. Static or dynamic ploughing lithography (dSPL), for example, utilizes the mechanical interaction between the tip and the resist, comparable to scratching [113–114]. In thermal SPL (tSPL) a heated AFM tip is used to evaporate the polymer [115–116]. In local anodic oxidation scanning probe lithography (oSPL) [117–118] a water meniscus is formed between the tip and the sample due to an applied voltage. Inside this water meniscus local oxidation of the sample takes place. The resolution is limited for dSPL by the tip size and tilting during the ploughing process, for tSPL by the heat diffusion and for oSPL by the size of the water meniscus and the oxidation reaction. Using tSPL and oSPL, features with sizes in the sub-10 nm range (dots) and pitches down to 15 nm (oSPL) can be generated [116–117].

Very soon after its invention the scanning tunneling microscope was used for lithography as well [119–120]. Here, the resist exposure occurs due to electrons tunneling between the tip and resist or substrate [121–123], which results in the disruption of chemical bonds, e.g., Si–H bonds for silicon surfaces. The method must be performed in vacuum but can in principle achieve atomic resolution [124]. Writing speeds for this process are up to 100 nm·s^−1^, which is similar to values achieved for scanning electron beam systems as described in Section 2.2 [9].

#### 3.1 Field-emission electron scanning probe lithography

**Figure 14:**
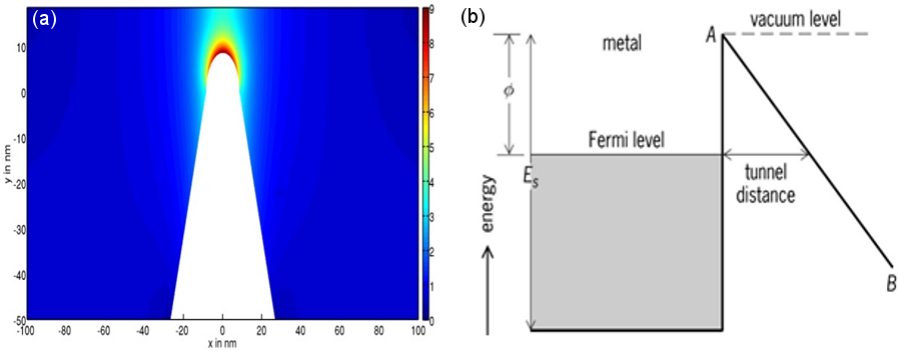
(a) Thermographic scale representation of the electric field between tip and sample calculated by solving the Laplace equation for a voltage of 50 V, a tip–sample distance of 10 nm and a tip diameter of 17 nm. (b) Schematic description of the fundamentals of electron tunneling into air from the tip of a scanning probe.

In this review, we will focus on field emission electron scanning probe lithography (FE-eSPL) as the chosen charged particle beam-based SPL method, which offers high-resolution lithography and high writing speeds [125–126]. In FE-eSPL, electrons emitted from the tip of a scanning probe are used to expose the resist. In this respect, the FE-eSPL process is similar to EBL but the energy of the primary beam used for SPL is much lower (below 100 eV) and the electron beam is generated in close proximity to the sample surface (below 100 nm), thus without the need for optics to control the beam. To trigger the electron emission a voltage is applied between sample and tip. Due to the tip shape the electric field is enhanced up to 25-times near the tip [127] and electrons are consequently emitted [128–130]. In Figure 14a, the electric field between tip and sample is shown for a representative tip shape and tip–sample distance. The physical process of emission is quantum mechanical tunneling of the electrons through the potential barrier, which is tilted due to the electric field (see Figure 14b for a schematic description of the tunneling process).

The theory of electron field emission from metals was developed by Ralph H. Fowler and Lothar Nordheim [131]. This process is governed by Equation 4:

[4]
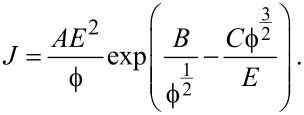


The equation describes the dependence of the emitted current density *J* on the electric field *E* and the work function 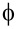 of the tip material. *A*, *B* and *C* are constants, which are given, e.g., in [132]. Since the electric field strongly depends on the tip diameter, tip–sample voltage and the tip–substrate distance, these can be varied in order to change the exposure current and current distribution [128,133].

The typical values for the tip–sample voltage are 30–100 V so that the energy of the electrons is in the range of 30–100 eV. This is relatively low in comparison with the primary beam energies of tens of kiloelectronvolts used in EBL. The typical tip–sample distance (<100 nm) is smaller than the mean free path of electrons in air at atmospheric pressure so there is no requirement for a vacuum system and the FE-eSPL process is carried out under ambient laboratory conditions. Furthermore, the energy range of the electrons generated in the FE-eSPL tool is very close to the binding energy of the resist molecules. The inelastic scattering of the electrons with the resist molecules, which drives the lithographic process, therefore has a strongly increased effect compared to that of the high-energy electrons typically used in EBL [134]. Therefore, the generation of secondary electrons with sufficient energy for the lithographic process is strongly reduced and backscattering of primary electrons at the sample surface [112,135–137]. Because of this, the proximity effect observed in EBL, which is caused by the generation and spread of the secondary electrons and by backscattering, is almost absent in SPL [138–139].

As in EBL, the electron exposure of the resist enhances or decreases the solubility of resist molecules, producing positive tone or negative tone structures [140]. A developer-free dry patterning process, only occurring for FE-eSPL, was discovered in 2003, first for calixarene resist [141] and later also for other resist materials [140,142–143]. In this tone the resist can be removed directly by sufficiently intense electron irradiation if the lithography takes place under ambient conditions. Unlike tSPL, FE-eSPL structuring is a cold process due to the low power density which has advantages when patterning over thin active device regions, which is common in R&D applications. In this respect, FE-eSPL avoids undesirable effects such as dopant spreading associated with local temperature rise.

**Figure 15:**
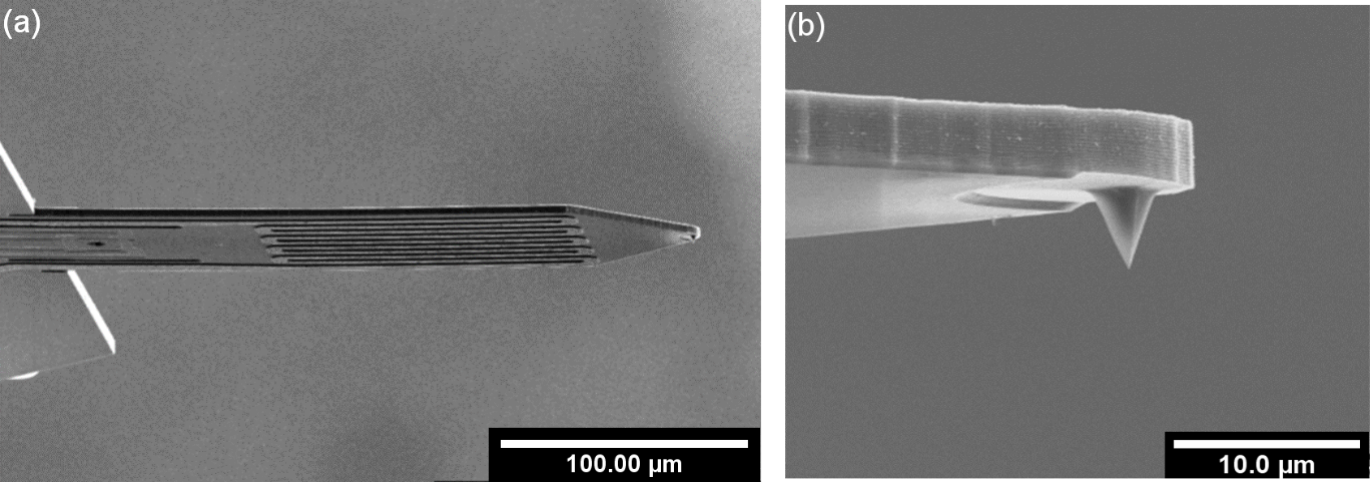
(a) SEM image of self-sensing and self-actuated cantilever with the thermomechanical actuator and piezoresistive deflection read-out. (b) Close-up SEM image of the tip at the front of the cantilever. Reproduced with permission through Creative Commons Attribution (CC BY) from [146], 2017 AIP Conference Series.

**Figure 16:**
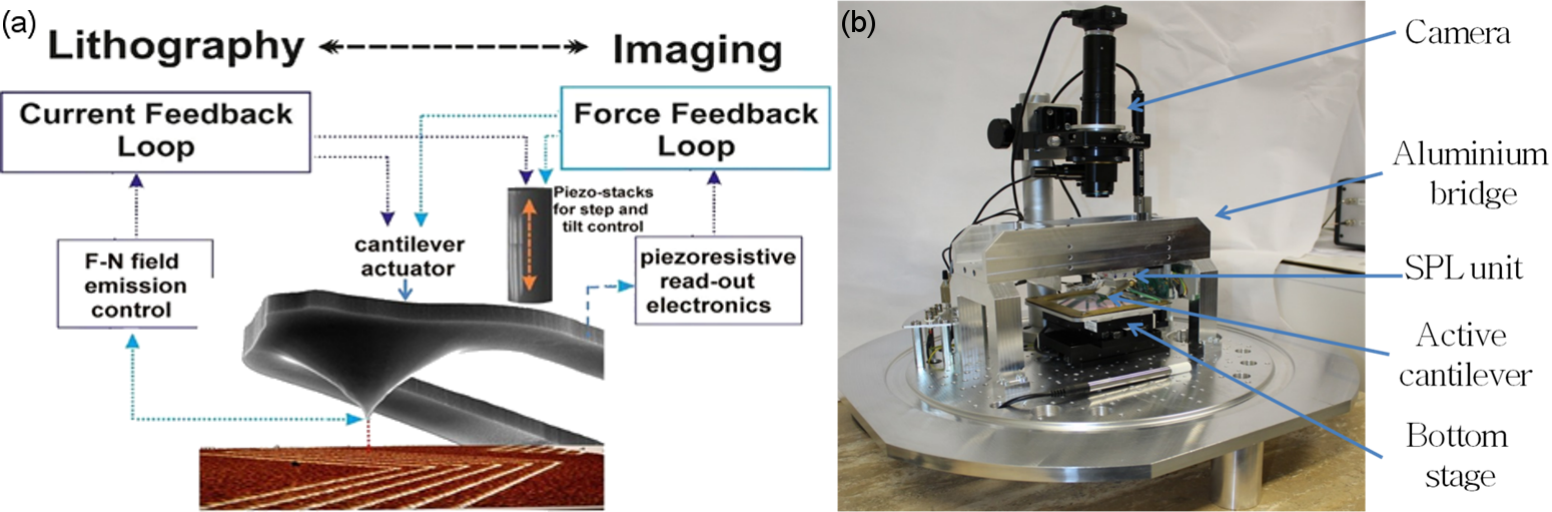
(a) Schematic showing feedback loops combined in FE-eSPL tool enabling lithography using the current feedback loop and measurement of written structures by AFM using the force feedback loop. Reprinted with permission from [157], copyright 2014 American Vacuum Society. (b) FE-eSPL tool developed in the Rangelow Group at TU Ilmenau. Reprinted with permission from [158], copyright 2018 Elsevier.

**3.1.1 Experimental set-ups forFE-eSPL.** A pattern is written by moving the scanning probe along a pre-defined path using a piezo-scanner [144]. FE-eSPL systems are typically operated in a constant-current mode [120,126], in which the height of the scanning probe, i.e., the tip–sample distance, is regulated to keep the current between tip and sample constant at a predefined current set-point. This takes advantage of the fact that the emission current depends on the electric field enhancement near the tip (Equation 4), which in turn depends on the distance between tip and sample. The hybrid STM/AFM system from Quate et al. [145] uses two simultaneously operating feedback loops: one to keep the tip–sample distance constant by measuring the cantilever deflection and adjusting the *z*-position of the scanner, and the second to maintain constant emission current by adjusting the tip–sample voltage. This system can be used in lithography mode or in AFM imaging mode employing typically passive cantilevers with optical read-out. The FE-eSPL developed in the Rangelow group (Figure 16) incorporates both modes as well, but uses so-called active cantilevers in cantilever scanning configuration [146]. These are self-actuated and self-sensing scanning probes [147], which can be used both for lithography and for measuring the generated structures by atomic force microscopy and related techniques such as Kelvin force microscopy or force–distance measurements, without the use of additional optical read-out. In Figure 15 an active cantilever is shown, together with a close-up view of the sharp tip at the end of the cantilever.

This active cantilever is equipped with a thermomechanical actuator and a piezo-resistive sensor for measuring the bending of the beam. The thermo-mechanical actuator is a loop made of a thin metal film of aluminium or aluminium–magnesium alloy [148] on top of the silicon beam. A current through the actuator results in heating that induces bending of the cantilever due to the bimorph effect. Thereby, a DC voltage generates a static deflection whereas an AC voltage can drive an oscillation of the cantilever. The deflection is determined by the heat generated resistively and up to 30 µm peak-to-peak values of the actuation amplitude can be achieved [149]. The deflection sensor comprises four piezo-resistors configured in an integrated Wheatstone bridge to reduce the influence of noise and temperature [150–151]. A simulation model was developed to optimize the size, length and placement of the heater and the deflection sensor [148,152]. Other actuation and sensing principles can be used as well [153]. The fabrication of the cantilevers uses a surface micromachining process in order to form sharp tips. Standard IC planar processing of silicon on insulator (SOI)-wafers is employed in combination with bulk micromachining to form the cantilever. Details about the fabrication process can be found in [154–155].

Atomic force microscopy relies on the actuation of the cantilever while simultaneously measuring the deflection [154]. Therefore, the tip–sample distance is regulated to keep the oscillation amplitude (amplitude-modulation AFM) or phase (phase-modulation AFM) at a pre-defined set-point. Since the oscillation amplitude depends on the force between tip and sample, its variation is a direct indicator for the topography and material properties of the sample. Tools that combine both feedback loops, i.e., current and force, are capable of both FE-eSPL and AFM using the same cantilever [145,156]. This is shown schematically in Figure 16 [157].

**Figure 17:**
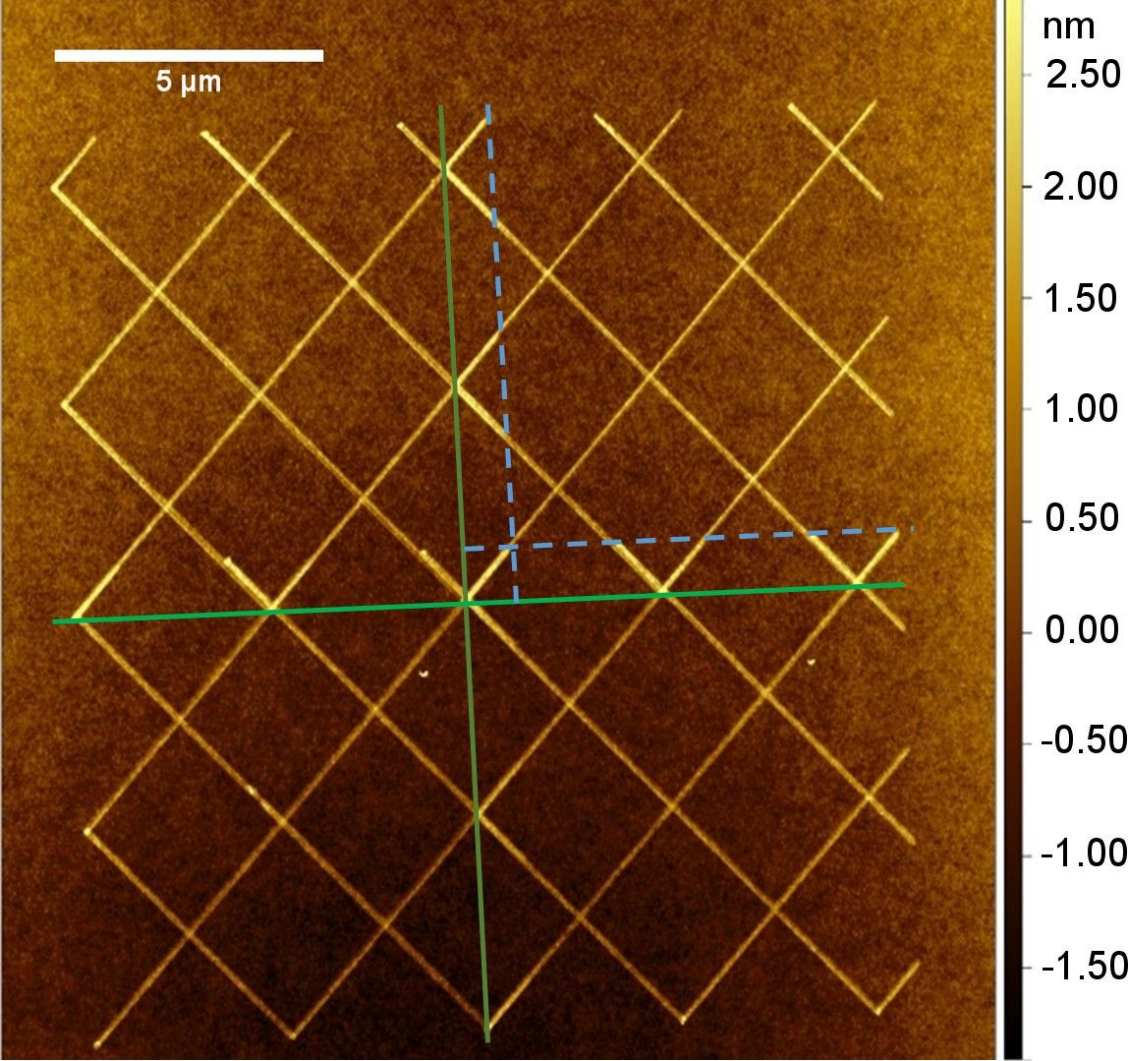
Example of stitching test showing an AFM image obtained directly after FE-eSPL exposure, showing negative tone features. The same pattern was written four times, indicated by the green lines, to compose the complete pattern. Stitching was achieved using scanner positioning. Before patterning the previously written features were measured with AFM and the new pattern was aligned to these structures, using an overlap of 0.5 µm with previous ones (indicated by the blue dashed lines).

Combination of FE-eSPL and AFM modes using active cantilever makes the setup capable of fast switching between modes. Thus, it enables (i) sample inspection before patterning, (ii) AFM-based registration, stitching and overlay alignment with high accuracy (ca. 10 nm using the bottom stage [159] and ca. 1 nm using the scanner positioning, see Figure 17), (iii) patterning by FE-eSPL and (iv) in situ inspection after each patterning cycle.

The FE-eSPL tool developed by the Rangelow Group at TU Ilmenau is shown in Figure 16. It can use two types of scanner for scanning probe movement and placement, providing either a 10 × 10 μm^2^ or 200 × 200 μm^2^ scan area. This top scanner is mounted on a cross-beam suspension made from aluminium or granite for high mechanical stability and low thermal drift. The use of a coarse positioning bottom stage, holding the sample, enables an increased patterning area of up to 100 × 100 mm^2^. The step-and-repeat function, used to generate the same structure repeatedly on the sample, enables an active pattern area over a 150 mm silicon wafer [160]. Optical navigation through a camera mounted on the top of the system enables coarse positioning.

**Figure 18:**
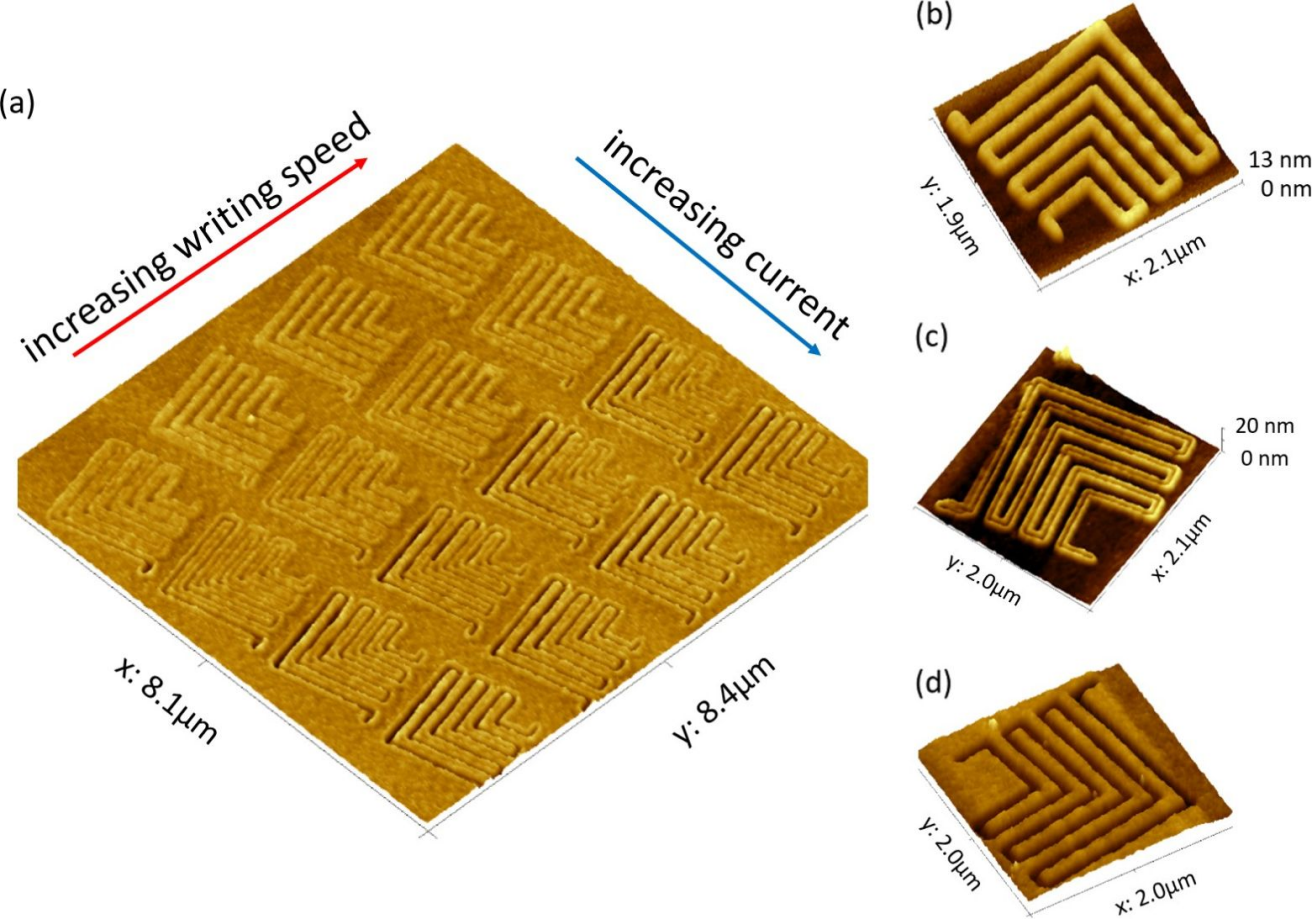
(a) Results of typical exposure dose test for determination of lithographic tone as a function of the exposure dose. AFM image of standard exposure dose test on resist AZ^®^ BARLi^®^ acquired directly after FE-eSPL. The dose is varied by changing the writing speed from 1 to 2.5 μm·s^−1^ (marked with red arrow) and by changing the current from 15 to 30 pA (blue arrow). (b–d) Close-up AFM image on different lithographic tones obtained after etching. (b) Negative tone feature, (c) intermittent tone, which is a combination of positive tone (trenches in the middle of the line) and negative tone (hills at the border of the lines), (d) positive tone features.

**3.1.2 Resists for FE-eSPL.** Monomolecular resists are favoured for FE-eSPL since they give smaller line edge roughness and higher resolution. The resist most commonly used in the FE-eSPL process is calixarene [126,161]. Calixarenes are macrocycles with molecule sizes of about 1 nm and are favoured as an FE-eSPL resist because of this small particle size, a low molecular weight, a precisely defined spatial extent, a monodisperse nature and a high uniformity. Furthermore, tone switching can be achieved with calixarene resists and other resists such as AZ^®^ BARLi^®^, when the exposure dose of the FE-eSPL process is increased [126], as shown in Figure 18. In addition, because of the high plasma etch durability of thin calixarene layers (<10 nm), essential for the production of nanodevices, calixarene resist is of considerable interest as a “prospective material for nanofabrication” [162].

**Figure 19:**
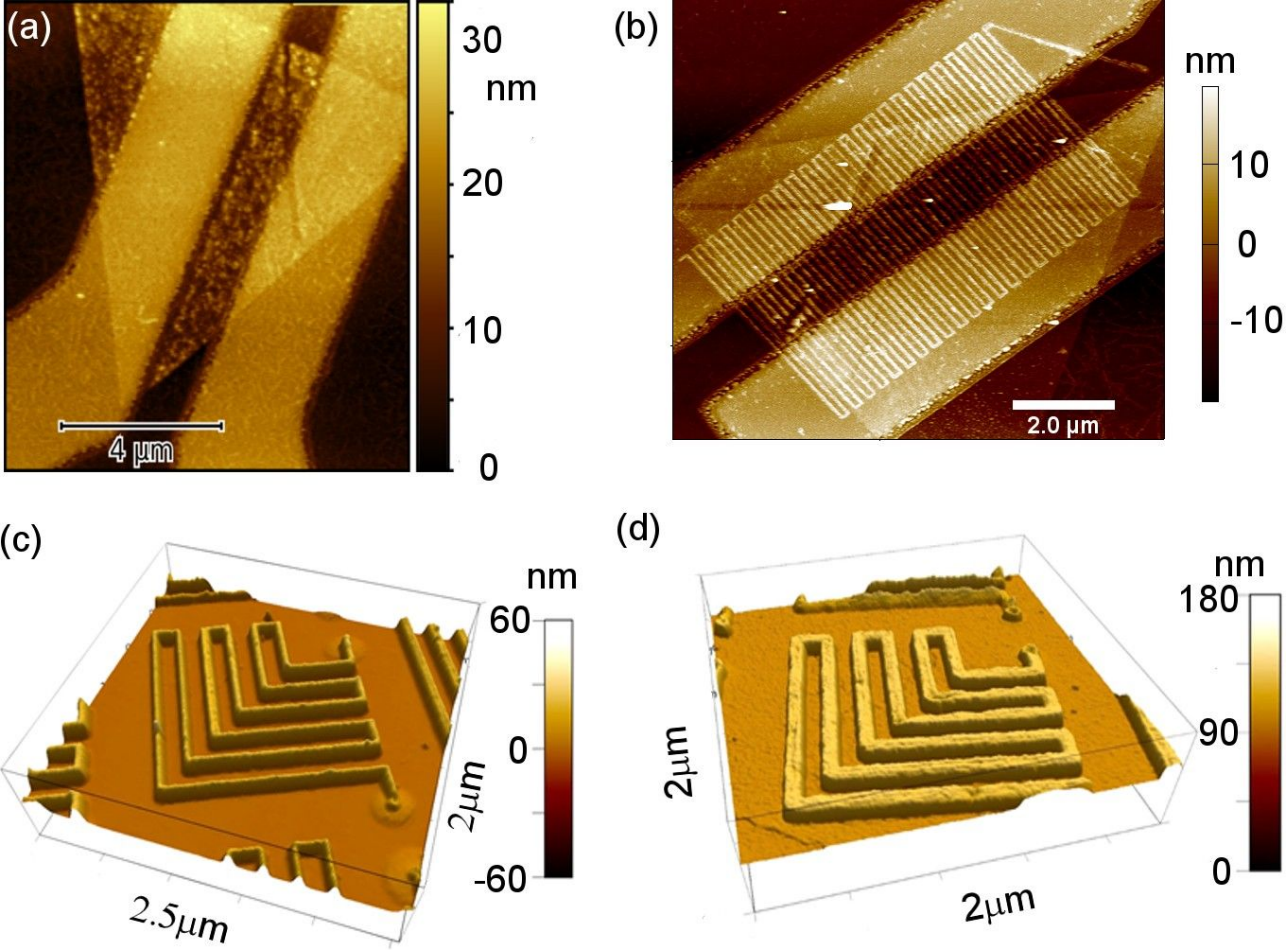
AFM images of FE-eSPL patterning of different materials. (a) MoS_2_-flake on SiO_2_ substrate placed between gold contacts before patterning. (b) MoS_2_ flake after FE-eSPL patterning and first development step. (c) Patterning demonstration of novel molecular glass resist UBT7 from University of Bayreuth. (d) Patterning demonstration of HM01 fullerene resist from Irresistible Materials Ltd.

While there is a wealth of experience with the molecular resist calixarene, other resists have been tested as well. For example, the FE-eSPL exposure was tested on standard EBL resists, namely poly(methyl methacrylate) (PMMA), polystyrene (PS) and polyhydroxystyrene (PHS) [140]. The development-less ablation mode could be obtained for all three resists as well as the positive tone for PMMA and the negative tone for PS and PHS after development. In comparison to EBL, a slightly higher electron dose is necessary in FE-eSPL for tone switching of PMMA resist and the sensitivity of PS is higher. Typically, the exposure dose for FE-eSPL differs from the doses for EBL and it is assumed that both methods address different exposure mechanisms or reaction pathways [138]. In particular, pathways in EBL using the generation of radicals are not possible for FE-eSPL due to the smaller energy of the incident electrons. However, the exact reaction pathways are still unclear. Plasma durability and FE-eSPL resolution were tested on the conductive resist poly(3-hexylthiophen-2,5-diyl) (short: P3HT), which can be used for insulating samples like glasses, since FE-eSPL always requires a conducting path at the sample. Tests on P3HT showed that standard FE-eSPL resolution of 10 nm half-pitch could be obtained and it exhibits plasma durability/etch stability similar to those of calixarene [163]. The use of P3HT enabled patterning on insulating substrates such as diamond or MoS_2_ flakes on SiO_2_ substrate (Figure 19a,b). For the latter, MoS_2_ flakes are actually conducting but due to their small thickness the current and the fast oxidation the conduction pathway for the emission current is quite unstable. Coating such samples with P3HT enables a stable FE-eSPL with high resolution as shown in Figure 19b. The samples were prepared at EPFL using exfoliation of MoS_2_ flakes onto a SiO_2_ substrate and subsequently adding gold contacts by EBL and lift-off. FE-eSPL patterning results in resist removal and direct oxidation of the MoS_2_ flake. Thereby, the precise regulation of the FE-eSPL tool allows patterning across topographical steps of 30 nm contact height without gaps or tip crashes (Figure 19b).

In addition, we investigated the use of novel fullerene resists and molecular glass resists for FE-eSPL [143,164–165]. These are experimental resists from the University of Bayreuth (UBT) and Irresistible Materials Ltd in conjunction with Oxford Scientific Consultants Ltd. UBT7 resist is a molecular glass resist built from aromatic benzenes and fluorine, which includes an X-shaped spiro-core unit and acts as negative tone resist. UBT8 resist has a structure similar to that of UBT7 but without the fluorine. Interestingly, the FE-eSPL structuring of UBT7 and UBT8 is different from the typically used calixarene resist regarding tone switching [143,164–165]. As described previously, the patterning tone can be switched from negative to positive for calixarene and other resists by increasing the exposure dose. For UBT7, in contrast, only negative tone is observed even for large exposure doses, while for UBT8 only positive tone patterning is seen [143].

The HM01 resist from IM Ltd is a novel negative tone spin-coatable liquid formulation based on the C_60_ fullerene molecule and has been discussed in Section 2 above. Figure 19c,d show results from FE-eSPL patterning in both UBT7 and HM01 resists. Both UBT7 and HM01 novel molecular resists have shown exceptional etch resistance in silicon etch trials conducted at the SNM partner institution IMEC. For example, HM01 from IM Ltd has an etch rate of less than 50 nm·min^−1^ in a Si etch plasma.

**Figure 20:**
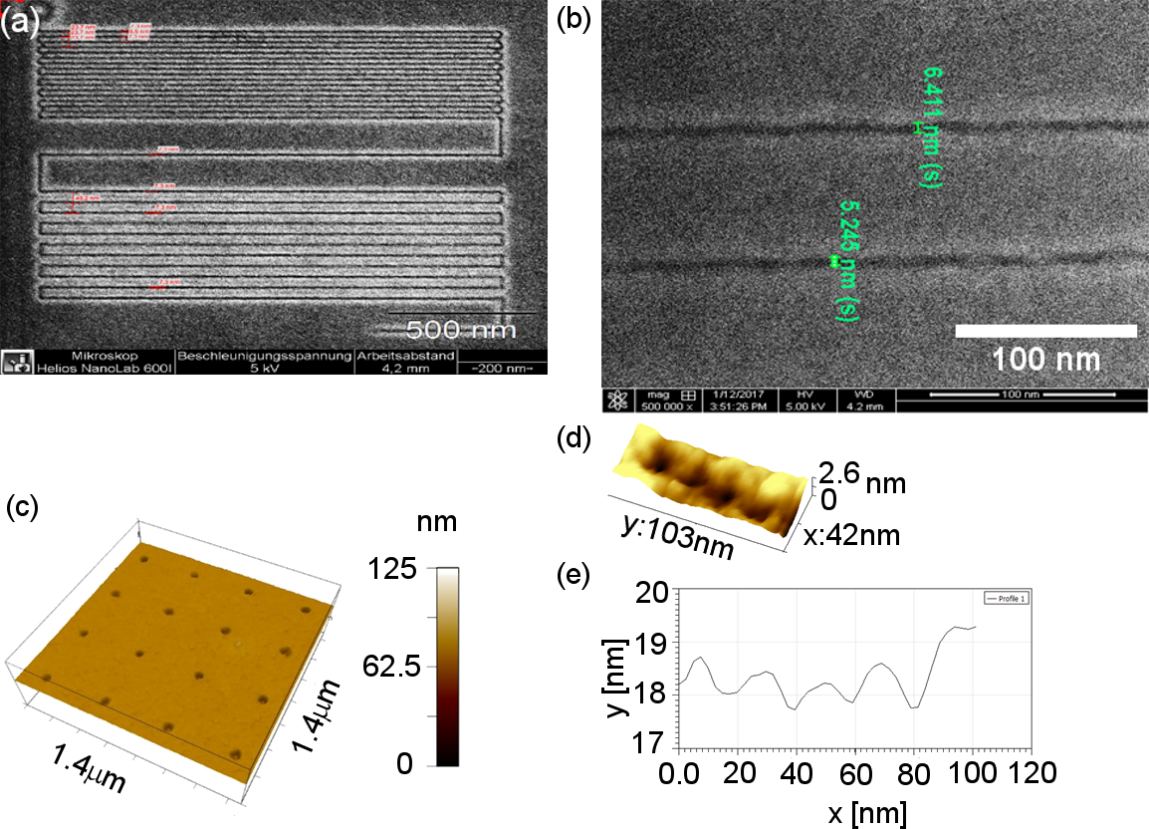
Single-digit nanometre features written by FE-eSPL. (a) SEM image obtained after etching of 9 nm thick AZ^®^ BARLi^®^ II resist (AZ Electronic Materials) showing 12.5 nm half pitch structures (upper part), (b) SEM image obtained after etching of 9 nm thick AZ^®^ BARLi^®^ II resist showing 5–6 nm thick lines, (c) AFM image after FE-eSPL exposure of P3HT showing regular 25 nm dot structures, (d) AFM image and (e) AFM profile obtained directly after FE-eSPL showing 7–10 nm (FWHM) dots in AZ^®^ BARLi^®^ II resist.

**3.1.3 Influences on FE-eSPL resolution.** The current state of the art in terms of the resolution of the FE-eSPL process is sub-5 nm line width (for single lines), 7.5 nm half pitch between two lines and sub-10 nm diameter for dot structures [126,143,160], as shown in Figure 20.

The FE-eSPL resolution is influenced by the tip shape and material, the exposure dose, the resist material, the tip–sample distance and the applied voltage. A variety of experiments and simulations were performed to elucidate the effects of the various influences. Since a smaller tip shape should result in smaller beam diameters [120], development of ultra-sharp tips is one route for enhancing the FE-eSPL resolution [166–167]. The tip–sample distance influences the electric field and thus the distribution of the emission current along the tip and the spread of the electrons in transit from the tip to the sample. Increasing the tip–sample distance results in larger beam diameters [132]. Decreasing the work function of the tip material while keeping the voltage constant results in larger beam diameters as well, since the distance between tip and sample is increased (in constant-current systems) [132].

The exposure line dose is *D*_l_ = *I*/*v* for current *I* and writing speed *v* of the scanning probe. Typically, currents in the range from 3–100 pA and writing speeds from 0.01 up to 10 μm·s^−1^ are used. Increasing the exposure dose results in larger structures [168–169], i.e., larger line widths or dot sizes, and in some resists in tone switching (from negative to development-less positive tone). To understand the influence of the exposure dose, it is important to differentiate between the different regulated modes of FE-eSPL systems. Systems operating in the constant-current and constant-voltage mode such as the Rangelow FE-eSPL tool adjust the tip–sample distance to maintain the current set-point. A change of the velocity therefore results in a decrease of the current distribution (smaller dose) but the shape of the distribution remains the same. In contrast, a change of the current set-point yields a change of the shape of the current distribution due to the modification of the tip–sample distance to reach the current set-point. For systems operating in the constant-current and constant-tip–sample-distance mode like the Quate FE-SPL tool, in which the voltage is adjusted to maintain the current set-point, changing the current set-point results in a change of the shape and maximal value of the current distribution due to the change of the applied voltage. Furthermore, changing the velocity in these systems requires adjustment of the applied voltage to maintain the same set-point [169]. This cannot be understood from the emission characteristics but was ascribed to the impedance change of the resist during exposure. The applied voltage scales the electric field and has thus, according to Equation 4, a strong influence on the current emission. For constant-current and constant-voltage systems, increasing the voltage results in an increased tip–sample distance and thus in an increased beam diameter [120,169].

Layer thickness and material of the resist influence the electric field and the interaction of the electrons with the resist. For increasing resist thicknesses, the electric field at the tip decreases as well as the energy of the electrons, when entering the resist layer [170–171]. Thus, for increasing thickness of the resist layer the distance between tip and resist surface should be decreased for the same current. The interaction of the electrons with the resist results in scattering and secondary-electron generation. This can increase the resulting line width in comparison with the current distribution of the electron beam on top of the resist.

Despite all these efforts, there remain unanswered questions about the FE-eSPL process. In particular, many simulations study the dependence of the beam diameter on the various parameters (e.g., tip shape, material, voltage and dose) but in the lithography experiments only the line width written into a resist can be measured. The conversion from beam diameter into line width has to take into account a dose or current threshold for structuring (resist and voltage dependent [172]), i.e., which dose or current is necessary to induce a resist change, and the scattering in the resist as well as the backscattering of electrons at the resist [173]. For example, a larger tip–sample distance can result in a larger spread of the emitted electrons (larger effective beam diameter) but simultaneously the maximum current density is reduced. In relation to the current density threshold for positive tone (ablation) or negative tone (cross-linking) behaviour, increasing the tip–sample distance may increase the line width due to the larger beam diameter, but if the current density maximum is close to the threshold a further increase will decrease the line width. Moreover, there are differences in the beam shapes extracted from experimental line widths, which are assumed to be Gaussian by Wilder et al. [138] and exponential by Mayer and Adams and co-workers [132,170].

**Figure 21:**
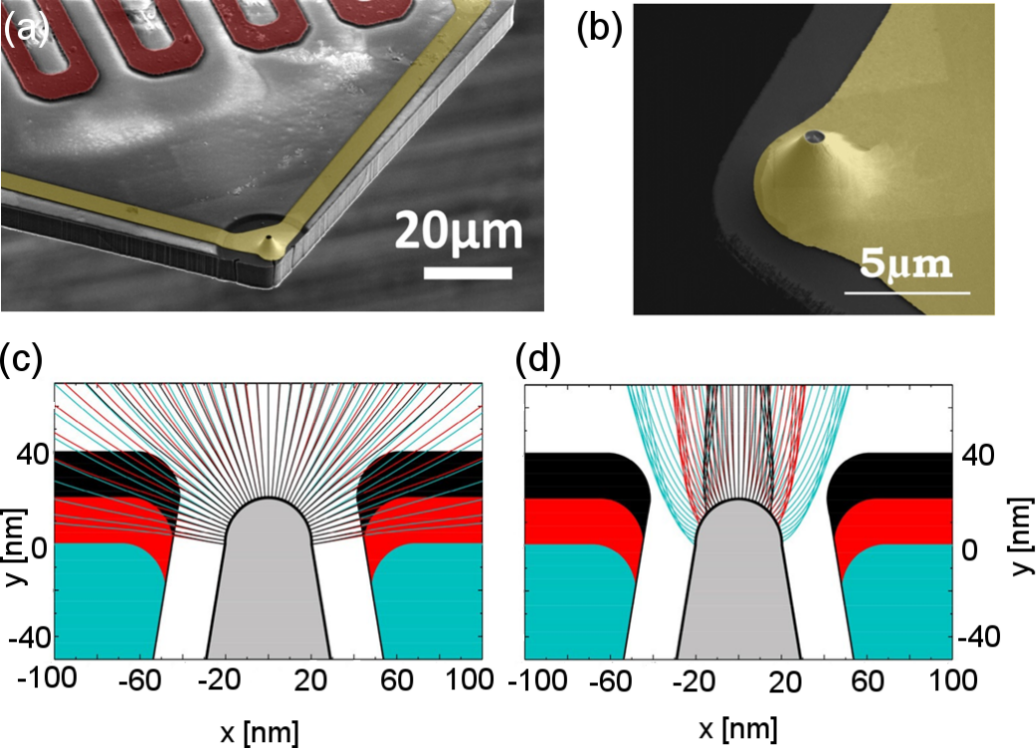
(a, b) SEM images of volcano-gated tip. (c, d) Simulation of the electron beam/trajectories for volcano-gated tips with different volcano heights showing the (c) defocusing and (d) focusing effects. Reproduced with permission from [175], copyright 2017 Elsevier.

**3.1.4 Simulation model for FE-eSPL.** To address these questions, a simulation tool was developed recently, which considers the main parts of the SPL process by calculating the electric field, the emission process, the trajectories of the electrons from the tip to the sample and the scattering processes inside the resist [174]. In this model, as a first step, the electric field between tip and sample is calculated based on the numerical solution of the Laplace equation. The electric field at the tip determines the emission probability of the electrons according to Equation 4. Thus, it strongly influences the number of emitted electrons – the lithography current – as well as the effective size of the electron beam. The electric field itself depends on the tip shape, the tip–sample distance and the voltage between tip and sample. The next step is the calculation of the electron trajectories from the tip to the sample since their spread sets the beam size at the sample. With this model, it could be shown that for each tip shape the external parameters (tip–sample distance, applied voltage, writing speed and current set-point) could be optimized to achieve maximum resolution. Thus, by adjusting tip–sample voltage, writing speed, tip–sample distance and exposure dose, the influence of fabrication-related tip-shape variations on the lithographic results can be reduced and the best resolution achieved. Since the trajectories are primarily affected by the electric field, shaping the electric field is a method to influence the FE-eSPL resolution. This was tested by using so-called volcano-gated tips, as shown in Figure 21 [175].

With a volcano-shaped electrode placed around the tip, changing the relation between the voltage at the gate and the voltage between tip and sample can be used to focus or defocus the electron beam [175]. The parameter range, in which focusing is achieved, is limited due to parasitic emission from the volcano gate. This can be overcome by using a gate material with a higher work function. Nevertheless, the complexity of the fabrication and the low resolution in AFM imaging of the volcano-gated tips, due to the larger effective interaction size (tip size plus volcano gate size) has limited their wide-spread use.

**Figure 22:**
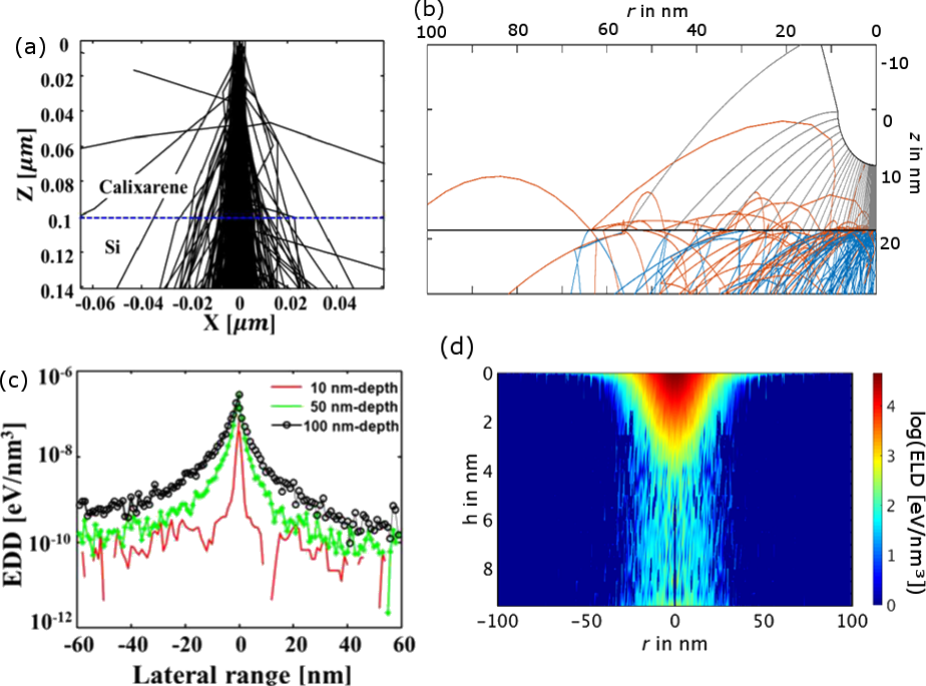
(a) Electron trajectories for 30 keV EBL exposure of 100 nm calixarene film on a Si sample. (b) Electron trajectories for FE-eSPL with 50V tip bias, 10 nm tip–resist distance and 10 nm thick calixarene resist (note that only one half of the tip and simulation area is shown). (c) Deposited energy distribution (EDD), describing the energy transferred from the electrons into the resist due to inelastic scattering, shown for various depths inside the resist for 30 keV EBL exposure of 100 nm calixarene film on a Si sample, for calculation details see [177]. (d) Energy loss distribution (ELD), describing the energy lost by the electrons due to inelastic scattering events, for 50 eV FE-eSPL exposure of 10 nm thick calixarene resist (note that the calculation differs from the EDD, thus giving different absolute values). (a, c) Images reproduced with permission through Creative Commons Attribution (CC BY) from [177], 2013 SciencePG; (b, d) images reproduced with permission from [174].

Finally, the interaction of the electrons with the resist is a major factor influencing the FE-eSPL resolution. This can be modelled using Monte Carlo methods [171], as described already for EBL [30]. The generation of secondary electrons and the inelastic mean free path play a major role in FE-eSPL resolution, which is below 1 nm for the FE-SPL process and around 10 nm in the EBL case (for 30 keV) [176]. This can be seen in Figure 22, where the trajectories of incoming and secondary electrons are plotted for 30 keV EBL exposure of a 100 nm calixarene resist layer (Figure 22a) and 50 eV FE-eSPL exposure of a 10 nm thick calixarene resist layer (Figure 22b; here, orange trajectories mark backscattered electrons). For EBL an almost continuous spread of trajectories with depth in the resist occurs, continuing in the Si sample, due to the large initial electron energy (Figure 22a), while in the FE-eSPL case a scattering can be observed mainly in the first layers of the resist (up to 2–3 nm, Figure 22b), since then the electrons lost almost all their complete kinetic energy due to scattering. Note, that for FE-eSPL the complete trajectories are plotted (continuing also for very small electron energies) while for EBL trajectories are typically plotted only for electrons with energies larger than a certain offset (typically 100 eV).

Focusing of the electrons in the resist due to the electric field in the FE-eSPL case could not be seen in the simulations, since the electric field in the resist layer is quite small. Despite the spread of the trajectories in the FE-eSPL case, the energy transfer into the resist by inelastic scattering is spatially limited to a small volume at the top of the resist layer due to the small inelastic mean free path and low energy of electrons. This can be seen in Figure 22d, where the distribution of the electron energy loss (ELD) due to ineleastic scattering is shown in dependence of the depth in the resist. A broadening of the incident beam is visible only for the first 2–3 nm and the size of the ELD remains smaller and almost constant with increasing depth in the resist. For EBL the interaction volume increases with increasing depth in the resist, which can be seen from the broadening of the distribution of deposited energy (EDD) in the resist with increasing depth into the resist (Figure 22c). Practically, the sensitivity and contrast behaviour of the resists have to be taken into account, also determining the cut-off energy for patterning.

**Figure 23:**
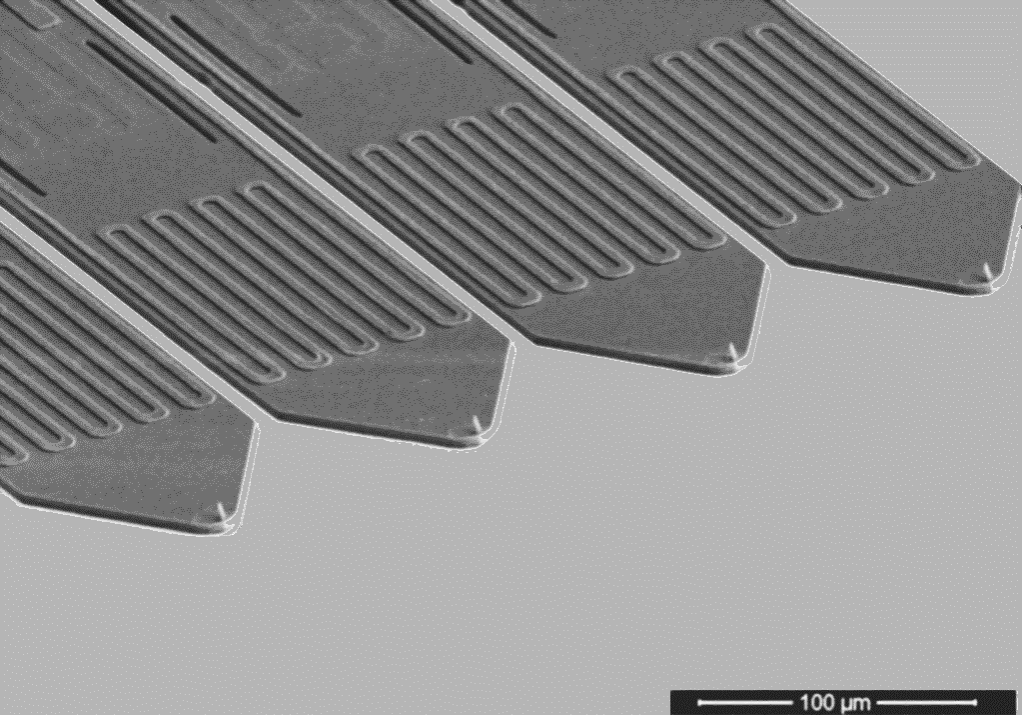
SEM image of Quattro cantilever array used for parallel AFM imaging with four cantilevers.

**3.1.5 Throughput enhancement.** Besides resolution, throughput is another important property for comparison of different nanostructuring methods. Here, three approaches are used: improvement of the FE-eSPL control algorithms, parallelization and combination with high-throughput techniques. Improving the control algorithms includes also faster electronics for the feedback loop as well as optimized scanner designs [144,169]. With regard to parallelization, active cantilevers including actuation and sensing enable the use of arrays with individually controlled cantilevers [156,178] and the “Quattro” cantilever array (Figure 23) was developed as well as the necessary control system [149].

With this system, the simultaneous acquisition of four AFM images was demonstrated, given a complete AFM image of 125 μm × 500 μm size and thus an effective scan speed of 5 mm·s^−1^ [149]. The same system can be used also for FE-eSPL and successful lithography with three of four cantilevers was demonstrated. The fourth cantilever could not successfully be used for lithography due to the large sample tilt in relation to the initial tilt of the cantilever array. For AFM imaging this can be overcome by applying a DC current to the thermomechanical actuation, which results in a static deflection of the cantilevers compensating the sample tilt or the array tilt. For FE-eSPL, stiffer cantilevers are used, so that, for the same DC current, a smaller static deflection of the cantilever is obtained, and thus this compensation mechanism cannot be applied. In the FE-eSPL case, tilt compensation is achieved using array holders or sample stages that can be tilted very accurately [149].

The combination of FE-eSPL with other lithographic techniques can increase the fabrication throughput. Hence, the “mix and match” approach can be used [126], in which large structures are generated by conventional optical lithography and only the high-resolution features are written with FE-eSPL. The applicability of this approach was shown for the fabrication of single electron transistors operating at room temperature [179]. Another approach is to use FE-eSPL for the fabrication of templates, which can be used for nanoimprint lithography (NIL). Here, the high-resolution features are fabricated by FE-eSPL on a stamp that can be reproduced with high fidelity and high throughput using NIL [180].

In conclusion, closed loop FE-eSPL [179] is a method (a) for the generation of high-fidelity lithographic features in positive and negative tone, and (b) for providing single-digit (sub-5 nm) resolution and highly accurate alignment capability as well as (c) step-and-repeat, multi-step and multi-layer lithography. Also, (d) it can be operated under ambient conditions as well as in vacuum. It can be used in mix-and-match lithography for high-throughput, e.g., for novel electronic devices, in particular in combination with cryogenic plasma etching, offering anisotropic and high resolution, and selective pattern transfer [163,181]. Scanning proximal tips can be employed as mechanical, thermal, chemical and/or electric sources to initiate and perform various physical or chemical lithographic processes. The ability to fabricate features reproducibly in the sub-5 nm regime enables the generation of nano-electronic and quantum devices [179]. FE-eSPL is a simple, reliable, cost-effective, and flexible technique to create patterns with nanometre resolution and is a promising candidate for fabrication of nanometre-scale devices and nanoimprint templates [180].

### 4 Conclusion

Three charged particle beam technologies for nano-patterning, namely scanning He^+^ ion beam lithography (SHIBL), focused electron beam induced processing (FEBIP) and field-emission electron scanning probe lithography (FE-eSPL) are reviewed against the historical background of electron beam lithography (EBL) with the latest results from the EU Project 318804 “Single Nanometre Manufacturing: Beyond CMOS”, which was completed in 2017.

SHIBL has many points of comparison with EBL but the use of the heavier He^+^ ions instead of electrons has advantages for nanometre-scale dense features because of the virtually zero proximity effect. In combination with a novel spin-coatable fullerene resist, SHIBL has achieved linewidths down to a continuity limit of 6 nm defined by shot noise. Its main use will be for circuit prototyping and potentially for nanoimprint lithography. It may also be used in mix-and-match with other lithographies including optical projection lithography.

The same is true of the other two lithography methods considered in this review. FEBIP has potential as a high-resolution lithography technique, extending EBL into the single-digit nanometer range. It uses focused electron beams without resist (in contrast to EBL) to deposit or etch with the assistance of chemical precursors dispensed from a nozzle close to the target surface. Significant throughput enhancement of FEBIP can be achieved using multiple beams in parallel and the current state of the art in this regard is 196 nanobeams developed at TU Delft on a scanning electron microscope platform. Large-area applications can be realized at high resolution using nanoimprint lithography and the manufacture of NIL templates is a major potential application of FEBIP. The required pattern transfer, to increase the aspect ratio of NIL templates, of EBID patterns into the template material through reactive plasma etch has been shown to be feasible in the sub-10 nm range.

The third charged particle lithography technology reviewed, FE-eSPL, is distinct from the other techniques in relying on a nanoscale physical probe maintained in controlled close proximity to the target surface. Developed from the scanning probe microscopy methods of AFM and STM, FE-eSPL typically has larger beam sizes, but smaller interaction volumes in the resist than EBL. The resolution can be tuned by varying external parameters such as tip voltage, tip–sample distance and exposure dose. A major advantage is that FE-eSPL is a cost-efficient technology that can be carried out under ambient conditions. It offers different reaction pathways and exposure mechanisms than EBL (different dose, development-less positive tone using calixarene resist). The integration of independently controllable self-actuated and self-sensing cantilevers in arrays, which enables lithography and imaging with high throughput is under development for potentially massive parallelization.

The benefits of using novel fullerene based molecular resists for the charged particle beam lithography methods discussed has become apparent from the work of the SNM project reported here, including significant resolution and plasma etch resistance advantages.

**Table 3:** List of abbreviations.

AFM	atomic force microscopy
BSE	backscattered electron
CMOS	complementary metal-oxide-semiconductor
CVD	chemical vapour deposition
EBID	electron beam induced deposition
EBIE	electron beam induced etching
EBL	electron beam lithography
EDD	deposited energy distribution
ELD	energy loss distribution
ESEM	environmental scanning electron microscope
EUV	extreme ultra violet
FE	field emission
FEBIP	focused electron beam induced processing
FE-eSPL	field-emission electron scanning probe lithography
FWHM	full width at half maximum
GFIS	gas field ion source
HIM	helium ion microscope
HM01	fullerene molecular resist from IM Ltd
HSQ	hydroxy silsesquioxane (resist)
IC	integrated circuit
IPA	isopropyl alcohol
LER	line edge roughness
LMIS	liquid metal ion source
MBSEM	multibeam scanning electron microscope
MEMS	microelectromechanical system
MIBK	methyl isobutyl ketone
NEMS	nanoelectromechanical system
NIL	nanoimprint lithography
PE	primary electron
PHS	polyhydroxystyrene
PMMA	poly(methyl methacrylate)
PS	polystyrene
P3HT	poly(3-hexylthiopen-2,5-diyl)
SE	secondary electron
SEM	scanning electron microscope
SHIBL	scanning helium ion beam lithography
SIBL	scanning ion beam lithography
SPL	scanning probe lithography
STM	scanning tunneling microscope
TEM	transmission electron microscope
TFE	thermal field emitter
UBT7, UBT8	molecular glass resists from University of Bayreuth
UHR	ultrahigh resolution
